# Deficiencies in the Mitochondrial Electron Transport Chain Affect Redox Poise and Resistance Toward *Colletotrichum higginsianum*

**DOI:** 10.3389/fpls.2019.01262

**Published:** 2019-10-17

**Authors:** Christopher McCollum, Sonja Geißelsöder, Timo Engelsdorf, Anna Maria Voitsik, Lars M. Voll

**Affiliations:** ^1^Division of Biochemistry, Department Biology, Friedrich-Alexander-Universität Erlangen-Nürnberg, Erlangen, Germany; ^2^Molecular Plant Physiology, Department of Biology, Philipps-University Marburg, Marburg, Germany

**Keywords:** Arabidopsis, *Colletotrichum higginsianum*, mitochondrial electron transport chain, alternative oxidase, metabolomics, redox signaling, cytochrome pathway

## Abstract

To investigate if and how the integrity of the mitochondrial electron transport chain (mETC) influences susceptibility of *Arabidopsis* toward *Colletotrichum higginsianum*, we have selected previously characterized mutants with defects at different stages of the mETC, namely, the complex I mutant *ndufs4*, the complex II mutant *sdh2-1*, the complex III mutant *ucr8-1*, and a mutant of the uncoupling protein *ucp1-2*. Relative to wild type, the selected complex I, II, and III mutants showed decreased total respiration, increased alternative respiration, as well as increased redox charge of the NADP(H) pool and decreased redox charge of the NAD(H) pool in the dark. In the light, mETC mutants accumulated free amino acids, albeit to varying degrees. Glycine and serine, which are involved in carbon recycling from photorespiration, and N-rich amino acids were predominantly increased in mETC mutants compared to the wild type. Taking together the physiological phenotypes of all examined mutants, our results suggest a connection between the limitation in the re-oxidation of reducing equivalents in the mitochondrial matrix and the induction of nitrate assimilation into free amino acids in the cytosol, which seems to be engaged as an additional sink for reducing power. The *sdh2-1* mutant was less susceptible to *C. higginsianum* and did not show hampered salicylic acid (SA) accumulation as previously reported for *SDH1* knock-down plants. The ROS burst remained unaffected in *sdh2-1*, emonstrating that subunit SDH2 is not involved in the control of ROS production and SA signaling by complex II. Moreover, the *ndufs4* mutant showed only 20% of *C. higginsianum* colonization compared to wild type, with the ROS burst and the production of callose papillae being significantly increased compared to wild type. This indicates that a restriction of respiratory metabolism can positively affect pre-penetration resistance of *Arabidopsis*. Taking metabolite profiling data from all investigated mETC mutants, a strong positive correlation of resistance toward *C. higginsianum* with NADPH pool size, pyruvate contents, and other metabolites associated with redox poise and energy charge was evident, which fosters the hypothesis that limitations in the mETC can support resistance at post-penetration stages by improving the availability of metabolic power.

## Introduction

Transition metal ions are frequently found as cofactors in enzymes. Their electron configuration renders transition metals particularly valuable as electron acceptors or donors in the catalysis of redox reactions. The photosynthetic electron transport chain (pETC) in chloroplasts and the respiratory electron transport chain (mETC) in mitochondria have special requirements for micronutrients like iron (Fe), zinc (Zn), copper (Cu), and manganese (Mn) (as reviewed by DalCorso et al., 2014). These metal ions are needed for structural components of the ETCs, predominantly as part of FeS clusters, but they also serve as cofactors in antioxidant enzymes like superoxide dismutases (SODs). SODs are integral elements of the soluble antioxidant system that has a strong buffer capacity to scavenge reactive oxygen species (ROS) that are generated as natural by-products of the ETCs in chloroplasts and mitochondria, but it also operates in peroxisomes, in the cytosol, and in the apoplasm to detoxify ROS in order to prevent oxidative damage to cellular enzymes and metabolites (Waszczak et al., 2018).

ROS are a group of low-molecular weight compounds that, by definition, show increased reactivity compared to molecular oxygen (O_2_). In plant cells, diverse forms of ROS occur, ranging from hydrogen peroxide (H_2_O_2_) and superoxide anions (O_2_^·−^) to hydroxyl radicals (^·^OH) and singlet oxygen (^1^O_2_) (Mignolet-Spruyt et al., 2016). In photoautotrophic leaf cells, ROS production in chloroplasts and peroxisomes is more than one order of magnitude higher than that from mitochondria (Foyer and Noctor, 2003). While considerable progress has been made in characterizing signaling events and outputs in response to singlet oxygen (^1^O_2_) and H_2_O_2_ generated in chloroplasts (op den Camp et al., 2003; Ochsenbein et al., 2006; Lee et al., 2007; Balazadeh et al., 2012; Kim et al., 2012; Sewelam et al., 2014), only comparatively little is known about how ROS generation in mitochondria is controlled, perceived, and communicated to the cytosol and the nucleus, with some reports being contradictory.

**Figure 1:**
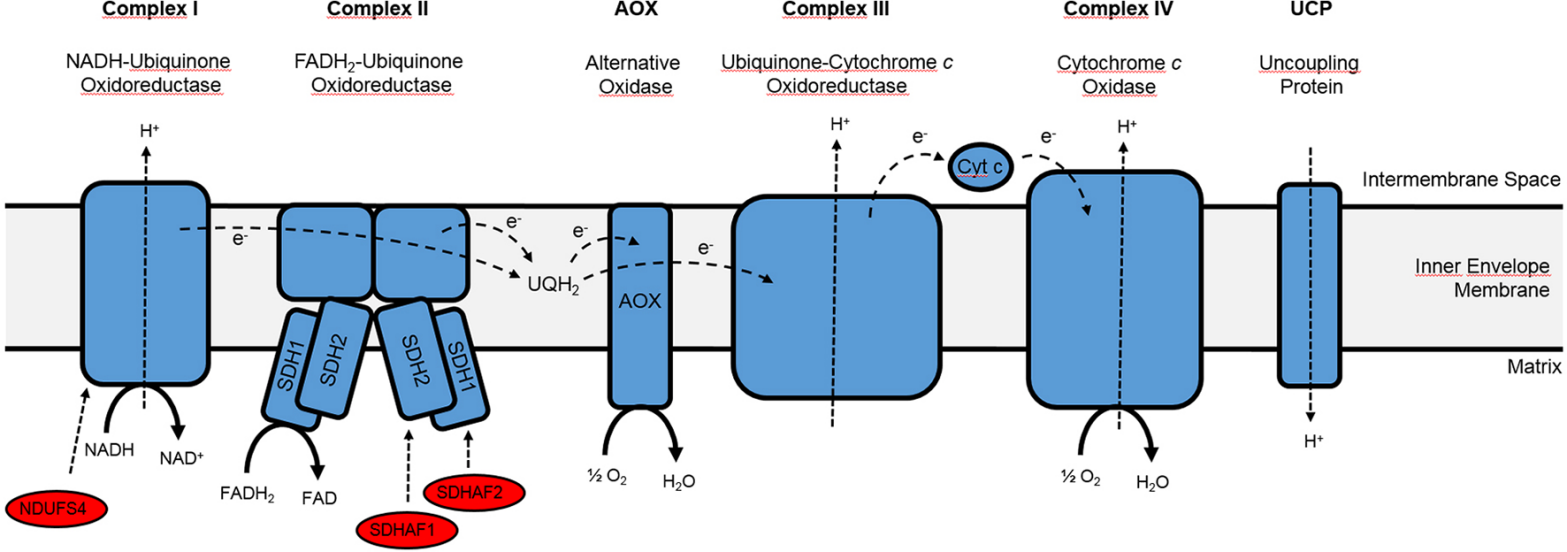
Simplified overview of the mitochondrial electron transport chain.Please note that only the complexes and assembly factors of special interest are depicted, while internal and external NAD(P)H dehydrogenases as well as complex V (ATP synthase) have been omitted. Cyt c, Cytochrome *c*; SDHAF1, succinate dehydrogenase assembly factor 1, which catalyzes the insertion of the Fe-S cluster into the SDH1 subunit of complex II; SDHAF2, succinate dehydrogenase assembly factor 2, which catalyzes the insertion of FAD into the SDH2 subunit of complex II; NDUFS4, complex I assembly factor NADH: ubiquinone oxidoreductase Fe-S protein4; UQH_2_, Ubiquinol. Adapted from Raghavendra and Padmasree (2003) and Huang et al. (2013).

The mETC is thought to represent the major source of ROS in animal and plant mitochondria (Huang et al., 2016), but functional studies from plant systems do not result in a clear picture. This is probably due to the fact that mETC complexes represent essential functions during germination and reproduction (see e.g., León et al., 2007; Huang et al., 2013; Kühn et al., 2015), which makes it difficult to isolate loss-of-function mutants for structural proteins of the respiratory complexes. Most of the characterized *Arabidopsis* mETC mutants carry weak alleles or mutations in assembly factors (Zsigmond et al., 2008; Meyer et al., 2009; Gleason et al., 2011; Huang et al., 2013; Kühn et al., 2015), which opens the possibility that electron transport and ROS generation are affected differently in mutants with a defect in the same respiratory complex. For an overview on components and assembly factors of the mETC that are of special interest for the following paragraph, please see **Figure 1**. For instance, no intact complex I was detected in the initial characterization of the *Arabidopsis* complex I assembly factor mutant *ndufs4* (*NADH:ubiquinone oxidoreductase Fe-S protein4*; Meyer et al., 2009), but growth and germination prove to be impaired much stronger in structural complex I mutants *ndufv1-1*, *ndufv1-2*, *ndufv2*, and *ndufv7* (*NADH:ubiquinone oxidoreductase flavoprotein1*) that were characterized in a later study (Kühn et al., 2015), indicating that the *ndufs4* mutant may contain trace amounts of complex I activity. Similarly, a point mutation in the complex II succinate dehydrogenase subunit *SDH1* gene resulted in 20% residual SDH activity of the corresponding *dsr1* (*disrupted in stress responses1*; Gleason et al., 2011) mutant and produced a much stronger physiological phenotype than in mutants lacking the complex II assembly factor SDHAF2, which assists to incorporate the FAD co-factor into mature SDH1 (Belt et al., 2017). While SDH activity was only reduced by 30% in *sdhaf2 (succinate dehydrogenase assembly factor2*) mutants, mitochondrial ROS production was decreased to a comparable extent as in *dsr1* under ADP-limiting state 4 conditions (Belt et al., 2017). Furthermore, only one of the two characterized *sdhaf2* T-DNA insertion alleles led to impaired seed development (Huang et al., 2013), which was also observed in heterozygous offspring of *sdh1-1* mutants with a comparable reduction in SDH complex activity (León et al., 2007). Interestingly, T-DNA insertion in the first exon of *SDHAF2* produced a milder phenotype compared to an insertion in the second intron of *SDHAF2* (Huang et al., 2013).

In general, ROS production by the mETC is positively correlated with the reduction state of its electron carriers (McDonald and Vanlerberghe, 2006). For instance, ROS production of isolated plant mitochondria increases when ADP is limiting and the membrane potential as well as the reduction state of the electron carriers concomitantly rise (McDonald and Vanlerberghe, 2006). Similarly, ROS generation increases upon the addition of the complex I inhibitor rotenone or the complex III inhibitor antimycin A that lead to an overreduction of certain sites of the mETC (Maxwell et al., 1999; Møller, 2001; Bernacchia et al., 2004).

Electron flow in the plant mETC is much more versatile than in animal mitochondria, since additional components like alternative oxidases (AOX), internal and external NAD(P)H dehydrogenases, as well as an uncoupling protein (UCP) can be engaged as endogenous uncouplers of electron transport and ATP production in unfavorable conditions, which results in diminished ROS production (McDonald and Vanlerberghe, 2006). Internal and external NAD(P)H dehydrogenases re-oxidize reducing equivalents by circumventing complex I and without building up the pH gradient, while AOX accepts electrons directly from ubiquinone (UQ) and transfers them onto molecular oxygen, thereby ameliorating the reduction state of complexes I, II, and III, which all donate electrons into the UQ pool. In contrast, UCPs directly release the overreduction of electron carriers by dissipating the membrane potential (Laloi et al., 1997). Interestingly, AOX and UCP seem to be regulated in response to ROS production. While AOX expression was shown to be induced by H_2_O_2_ (Vanlerberghe and McIntosh, 1996), UCP was found to be induced by O_2_^·−^ and lipid peroxidation products caused by O_2_^·−^ production (Considine et al., 2003; Smith et al., 2004).

Despite the clear correlation between reduction state of electron carriers and ROS production of isolated mitochondria *in vitro*, increasing ROS generation by the mETC *via* the application of antimycin A did not result in an elevated oxidation state of the mitochondrial matrix, as probed by redox-sensitive ro-GFP (Schwarzländer et al., 2009). Furthermore, the specificity and applicability of the most commonly used colorimetric and fluorescent dyes to probe ROS *in vivo*, like diaminobenzidine, Amplex Red, or dichlorofluorescein is under debate (Huang et al., 2016), making it difficult to assess the role of mitochondrial ROS in specific experimental conditions.

ROS are involved in regulating the response of plants toward pathogen infection in several ways (Lehmann et al., 2015). In close ties with Ca^2+^ signaling, ROS act as secondary messenger in biotic and abiotic stress situations, e.g., by the activation of NADPH oxidase RBOHD after binding of Ca^2+^ and calcium-dependent kinase 5 (CPK5)–dependent phosphorylation in *Arabidopsis* (Kimura et al., 2012). On the other hand, the recognition of pathogens by pathogen-associated molecular patterns (PAMPs) activates the oxidative burst, during which NADPH oxidases and class III peroxidases are activated in the apoplast (Mignolet-Spruyt et al., 2016). ROS formation during the PAMP-triggered oxidative burst is predominantly achieved by apoplastic peroxidases (Daudi et al., 2012). These peroxidases can oxidize target molecules in expense of H_2_O_2_ and subsequently generate O_2_^·−^ (Shigeto and Tsutsumi, 2015), while NADPH oxidases consume cytosolic NADPH to reduce O_2_ to O_2_^·−^ in the apoplast.

In addition to triggering the oxidative burst in the apoplast, recognition of PAMPs also activates ROS generation in chloroplasts (e.g., Zurbriggen et al., 2009). It has been shown that this can be achieved by reducing non-photochemical quenching (NPQ) in the PSII antenna (Göhre et al., 2012), which concomitantly increases ROS production by the pETC. Recent work of our group has revealed that increased chloroplastic H_2_O_2_ production activates WRKY33-dependent synthesis of indolic phytoalexins, which improves resistance of *Arabidopsis* toward the hemibiotrophic fungus *Colletotrichum higginsianum* (Schmidt et al., under review). The chloroplast-localized protein accelerated cell death2 (ACD2) serves in two cell organelles to balance ROS production and to counteract hypersensitive cell death (HR). Upon challenge with *Pseudomonas syringae*, ACD2 partially re-localizes from chloroplasts to mitochondria (Yao and Greenberg, 2006), and it detoxifies red chlorophyll catabolite (RCC) to non-fluorescent chlorophyll catabolites (NCCs) in both organelles, thereby preventing ROS formation and programmed cell death (Pattanayak et al., 2012).

The work on ACD2 shows that ROS formation in mitochondria represents an important regulator for HR. Although it has been proven experimentally that ROS generation in complex I and complex III mutants is increased (Zsigmond et al., 2008; Meyer et al., 2009), these mutants have not yet been tested for their interaction phenotype with pathogens. Interestingly, a point mutation in the complex II subunit gene *SDH1-1* conferred reduced ROS production from mitochondria in the *dsr1* mutant, which was identified in a screen for reduced responsiveness toward the defense messenger salicylic acid (SA) (Gleason et al., 2011), which also acts as a positive regulator of HR. This complex II mutant showed enhanced susceptibility toward virulent *P. syringae*, as well as against fungal leaf and root pathogens like *Alternaria brassicicola* and *Rhizoctonia solani* (Gleason et al., 2011). Using two independent complex II mutants in a later study, it was revealed that SDH activity and concomitant H_2_O_2_ production are stimulated by low concentrations of SA and that, in turn, increased SDH activity is required to activate SA-dependent reporter gene expression (Belt et al., 2017).

Taken together, the study of mutants has not revealed a clear relationship between hampered electron flow through individual complexes of the mETC and ROS production *in vivo*, and how these changes may affect the interaction with pathogens. However, extensive metabolic changes in TCA cycle, amino acid, and redox metabolism were commonly observed in mETC mutants (Dutilleul et al., 2005; Meyer et al., 2009; Gleason et al., 2011; Huang et al., 2013; Kühn et al., 2015; Belt et al., 2017). In the present study, we address the question, if certain metabolic patterns caused by defects in respiratory metabolism can affect compatibility. We chose to investigate this question in the interaction of *Arabidopsis* with the hemibiotrophic fungus *C. higginsianum*, since penetration and immediate post-penetration establishment of the pathogen seem to be quite insensitive to ROS (Birker et al., 2009). Leaf infections with *C. higginsianum* are initiated by conidia that land on the leaf surface and produce germ tubes, which then differentiate dome-shaped appressoria (O’Connell et al., 2004; Engelsdorf et al., 2013). Without delay, appressoria form penetration pegs that pervade the underlying epidermal cell wall predominantly by mechanic force (Bechinger, 1999; Deising et al., 2000) and establish bulbous infection vesicles and lobed biotrophic primary hyphae within the first 36h after inoculation (hpi), leaving the penetrated epidermis cells alive. At about 72 hpi, hyphal morphology, and lifestyle changes, necrotrophic hyphae emanate into adjacent cells and colonize the infected tissue rapidly by actively killing the encountered cells, leading to the formation of visible water-soaked lesions (Engelsdorf et al., 2013).

## Material and Methods

### Plant Material and Growth Conditions

Wild-type Col-0, alternative oxidase overexpressors (AOX1A-OX, Umbach et al., 2005), and alternative oxidase antisense lines (AOX1A-as; Umbach et al., 2005), as well as *ndufs4* (SAIL_596_E11; Meyer et al., 2009), *sdh2-1* (SALK_031100; Elorza et al., 2004), *ucp1-1* (SAIL_242_A09, carrying an identical T-DNA integration site compared to SAIL_536_G01, which was characterized by Sweetlove et al., 2006), and *ucr-8-1* (SALK_022835 with the T-DNA insertion predicted to be located at the end of the first exon of At3g10860) were obtained from NASC (Nottingham, UK), and homozygous offspring was identified by PCR as described in the respective publications. For genotyping the offspring of SALK_022835 (*ucr-8-1*), oligonucleotides SALK_022835-LP (5’-TGA GCA TAC CTG CAT TCA TTG-3’) and SALK_022835-RP (5’-TTG TTG TTG GGA TTA TCT GGG-3’) were used together with LBb1 (5’-GCG TGG ACC GCT TGC TGC AAC T-3’). Genotyping results for six representative *ucr8-1* individuals are shown in **Figure S1**. In addition to genotyping, AOX1A transcript levels in AOX1A-OX and AOX1-as were determined by qRT-PCR as described in Umbach et al. (2005), and *UCR8* transcript levels in *ucr8-1* were quantified relative to *ACTIN2* as described below (for results, please see **Figure S2**). All genotypes are in the Columbia (Col0) background.

*Arabidopsis thaliana* plants were grown in pots containing 65% (v/v) soil (Gebr. Patzer KG, Sinntal-Jossa, Germany), 25% (v/v) sand, and 10 (v/v) Liadrain expanded clay (Liapor, Pautzfeld, Germany). After stratification for 48h at 8°C, seeds were germinated for 2 weeks under short-day conditions [8h light (22°C)/16h darkness (19°C)] at 50 µmol quanta m^−2^ s^−1^ and were then cultivated for 3 more weeks in a growth cabinet under a 12-h light/12-h dark cycle at 22°C day/20°C night temperatures at a PFD of 90 µmol quanta m^−2^ s^−1^, before leaves were sampled for physiological analyses or challenged with 10^6^ C. *higginsianum* spores/ml by spray infection (see paragraph below).

### Quantitative RT-PCR to Determine *UCR8* Transcript Levels in *ucr8-1*

Total RNA from Col-0 and *ucr8-1* seedlings was extracted with a NucleoSpin RNA Plant and Fungi Kit (Macherey-Nagel), and 2 µg of total RNA were treated with RQ1 RNase-free DNase (Promega) and processed with the GoScript Reverse Transcription System (Promega). qRT-PCR was performed on a Bio-Rad MiniOpticon system using PowerUp SYBR Green Master Mix (Applied Biosystems). The following primer sequences have been used to analyze amplicons in *UCR8* (exon 1, E1; exon 1/exon 2, E1term/E2start; exon 2, E2) and *ACTIN2* (*ACT2*):

UCR8_E1-FW (5 ′-GTCTCTGGAAGGATCTGCCG-3 ′),
UCR8_E1-REV (5 ′-GGGGTAACGAGAAGAGTGGC-3 ′),
UCR8_E1term-FW (5 ′-ACAAGGTCTCTGAGAATTGGATCA-3 ′),
UCR8_E2start-REV (5 ′-TTCTCCTGTTCCTTGAAATACTGA-3 ′),
UCR8_E2-FW (5 ′-GTATGCTCAGTATTTCAAGGAACA-3 ′),
UCR8_E2-REV (5 ′-GAAAAGGAGTGACTTCATGCT-3 ′),
ACT2-FW (5 ′-CTTGCACCAAGCAGCATGAA-3 ′),
ACT2-REV (5 ′-CCGATCCAGACACTGTACTTCCTT-3 ′).

The normalized relative transcript quantity was determined as described by Rieu and Powers (2009).

### *C. higginsianum* Infection Assays and Assessment of leaf Colonization by *C. higginsianum*

Infection assays with the *C. higginsianum* isolate MAFF 305635 were essentially conducted as described by Voll et al. (2012).

Fungal colonization per leaf area was determined by qPCR during the necrotrophic stage as described by Engelsdorf et al. (2013).

### Characterization of Foliar Respiration

Dark respiration of fully expanded, slice-cut leaves from 5-week-old plants was determined with Hansatech oxygen electrodes as described by Umbach et al. (2005), except that 100mM KCN or 200mM SHAM were used to inhibit the cytochrome and alternative pathway, respectively, and that respiration rates were followed for 30 min before and after the addition of inhibitor. To obtain reliable quantitative data, respiration rates were recorded on oxygen electrodes that were frequently re-calibrated with 0% O_2_ and 21% O_2_, and calculated respiration rates were finally normalized to leaf fresh weight.

### Quantitative Determination of the ROS Burst Capacity

The quantitative determination of transient production of ROS in response to 2 µM flg22 was conducted according to Keppler et al. (1989). The ROS burst was determined from plants grown in three independent batches. Per batch, duplicate to triplicate experiments, each comprising eight leaf punches per genotype taken from independent, fully expanded leaves was measured and normalized to the respective wild-type value. The displayed data represent the average from all three independent batches, i.e., from a total of 56 samples obtained from three independent plant populations.

### Incidence of Callose Papillae at Attempted Penetration Sites

The incidence of callose papillae underneath fungal appressoria was quantified 2 dpi as described by Voll et al. (2012).

### Quantification of Salicylic Acid and Camalexin

Free SA and camalexin were determined following the procedure published by Voll et al. (2012).

### Metabolite Quantification

With the exception of nicotinamide adenine dinucleotides, all metabolites were determined as described in Horst et al. (2010), using four biological replicates of approx. 50-mg fresh weight from fully expanded leaves sampled 1 h before the end of the light period.

Nicotinamide adenine dinucleotides were quantified by cycling assays essentially as described by Kolbe et al. (2005), using 100-mg leaf material sampled 1 h before the end of the light period.

### Multivariate Data Analysis

Principle component analysis of normalized metabolite data was performed with the MarkerView Software (version 1.1.0.7, Applied Biosystems, Foster City, CA) using the Pareto algorithm for scaling. Values of the wild-type Col-0 were used as a reference for normalization.

### Statistical Analysis

Significance of pairwise differences was determined according to the Student’s *t*-test. Significance levels are indicated in the figure legends.

## Results

### Modulation of AOX1A Expression Does Not Seem to Affect the Interaction With *C. higginsianum*

**Figure 2:**
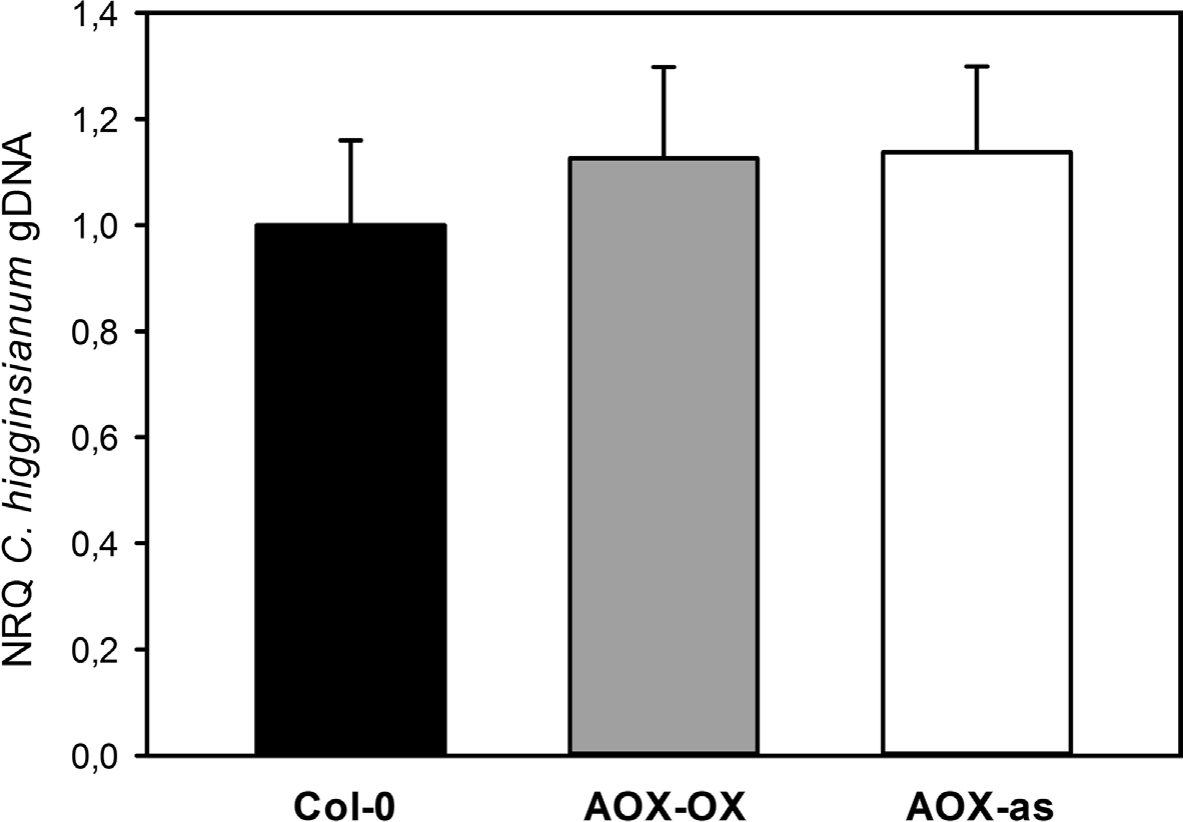
Modulation of AOX expression does not affect compatibility toward *Colletotrichum higginsianum*. Five-week old plants grown in 12-h light/12-h dark cycles were spray infected with 2·10^6^ *Colletotrichum higginisanum* conidiospores/ml 1 h before the end of the light period. *C. higginsianum* genomic DNA was quantified in infected, fully expanded *Arabidopsis thaliana* leaves by qPCR at 3.5 days postinfection (dpi) and is given as normalized relative quantity (NRQ) relative to Col-0. Col-0 (black bar), AOX-OX (gray bar), and AOX-antisense (white bar). The experiment was repeated eight times for Col-0 and AOX-OX and four times for AOX-antisense, and depicted data represents the average over all experiments ± SE. Per individual experimental replicate, data are means of four biological and three technical replicates per genotype and treatment.

As outlined in the introduction, limitations in the mETC can affect mitochondrial ROS production. While apoplastic and plastidic ROS are involved in pathogen defense, it is yet unclear if mitochondrial ROS can affect host-pathogen compatibility. A negative correlation of ROS production with AOX activity had previously been observed in tobacco protoplasts and intact *Arabidopsis* leaves with overexpression and antisense inhibition of AOX1 (Maxwell et al., 1999; Umbach et al., 2005). The difference between the genotypes was strong upon inhibition of the cytochrome pathway but still detectable in the absence of inhibitor (Maxwell et al., 1999; Umbach et al., 2005). Therefore, we investigated if *Arabidopsis* plants with antisense repression and overexpression of the main alternative oxidase isoform AOX1A (Umbach et al., 2005) exhibit altered compatibility toward *C. higginsianum*, but both genotypes behaved similar to wild type (**Figure 2**). Since it is unclear how individual defects in the mETC affect global ROS production and the interaction with leaf pathogens, we selected mutants for our study, in which electron flux through the alternative pathway should be increased in expense of the cytochrome pathway.

### Selection of Mutants With Increased Alternative Respiration

**Figure 3:**
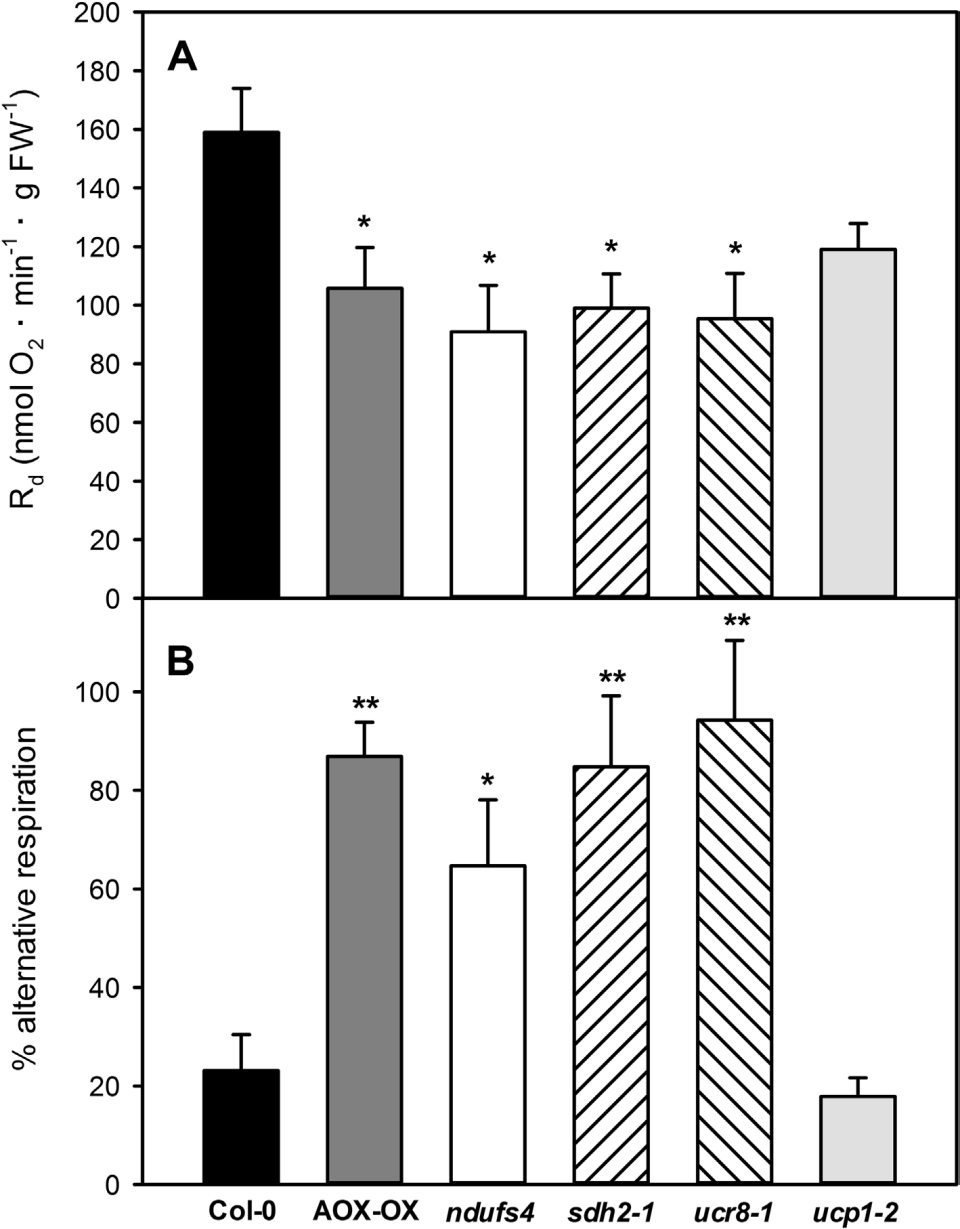
Dark respiration in mETC mutants. Dark respiration was determined in leaf strips of fully expanded leaves from 5-week-old plants according to Umbach *et al*. (2006), using a Clarke-type oxygen electrode. Dark respiration R_d_ **(A)** was determined as oxygen consumption over a period of 30 min after 30 min of initial acclimatization. The percentage of alternative respiration **(B)** was determined by recording KCN-insensitive respiration for additional 30 min after the addition of 100mM KCN, or until steady state was reached. Data are means of four independent biological replicates ± SE. Col-0 (black bars), AOX-OX (dark gray bars), *ndufs4* (white bars), *sdh2-1* (left hatched bars), *ucr8-1* (right hatched bars), *ucp1-2* (light gray bars). Significant differences to Col-0 in a pairwise t-test are indicated by asterisks with *: p < 0.05 and **: p < 0.005.

For our mutant portfolio, we selected mutants defective in different complexes of the mETC and prioritized those that had already been characterized in detail on the physiological and molecular levels. We picked the complex I mutant *ndufs4* (Meyer et al., 2009), the complex II mutant *sdh2-1* (Elorza et al., 2004), the previously uncharacterized complex III mutant *ucr8-1* (SALK_022835), carrying a T-DNA insertion in subunit 8 of the cytochrome bc_1_ complex (At3g10860), and a mutant of the uncoupling protein *ucp1-1* (Sweetlove et al., 2006). Quantitative transcript analysis by qRT-PCR revealed that *ucr8-1* (SALK_022835) is a knock-down mutant, in which less than 10% of wild-type transcript level is present (**Figure S2**). Analysis with three independent primer pairs covering amplicons in exon 1, exon 2, and at the exon 1–exon 2 junction resulted in highly consistent results, which suggests that *UCR8* transcripts produced from the *ucr8-1* allele are spliced correctly and that the T-DNA insertion site resides in the first intron of *UCR8* (**Figure S2**). Total respiration of the newly identified *ucr8-1* knock-down mutant was reduced by 40% (**Figure 3A**), which is comparable to what was observed for the previously characterized complex III mutant *ppr40-1* (Zsigmond et al., 2008). In comparison to wild type, we also observed a similar reduction in overall dark respiration by around 40% in the mutants *ndufs4* and *sdh2-1* (**Figure 3A**).

We were then interested if respiration through the alternative pathway is increased in the selected mutants by determining KCN-resistant respiration in dark adapted leaf strips. The wild-type Col-0 showed 23% KCN-resistant respiration compared to 87% in the AOX-OX overexpressors, which were used as a positive control (**Figure 3B**). Similarly, KCN-resistant respiration was increased to 84 and 94% in the complex II mutant *sdh2-1* and the complex III mutant *ucr8-1*, respectively (**Figure 3B**). The substantial increase in electron flux through the alternative pathway indicates that complex III function is strongly affected in *ucr8-1*. Similarly, alternative respiration was also substantial increased in the previously characterized complex III mutant *ppr40-1* (Zsigmond et al., 2008). In the complex I mutant *ndufs4*, 65% of the dark respiration were KCN-resistant (**Figure 3B**). Due to the presence of internal and external NAD(P)H dehydrogenases, total electron flux through the cytochrome pathway might be less affected in *ndufs4* compared to the complex II and III mutants. In contrast to the other three mutants, KCN-resistant dark respiration in *ucp-1-2* was comparable to wild type (**Figure 3B**).

### The Selected Mutants Show Individual Effects on NADP(H) and NAD(H) Pool Composition

**Figure 4:**
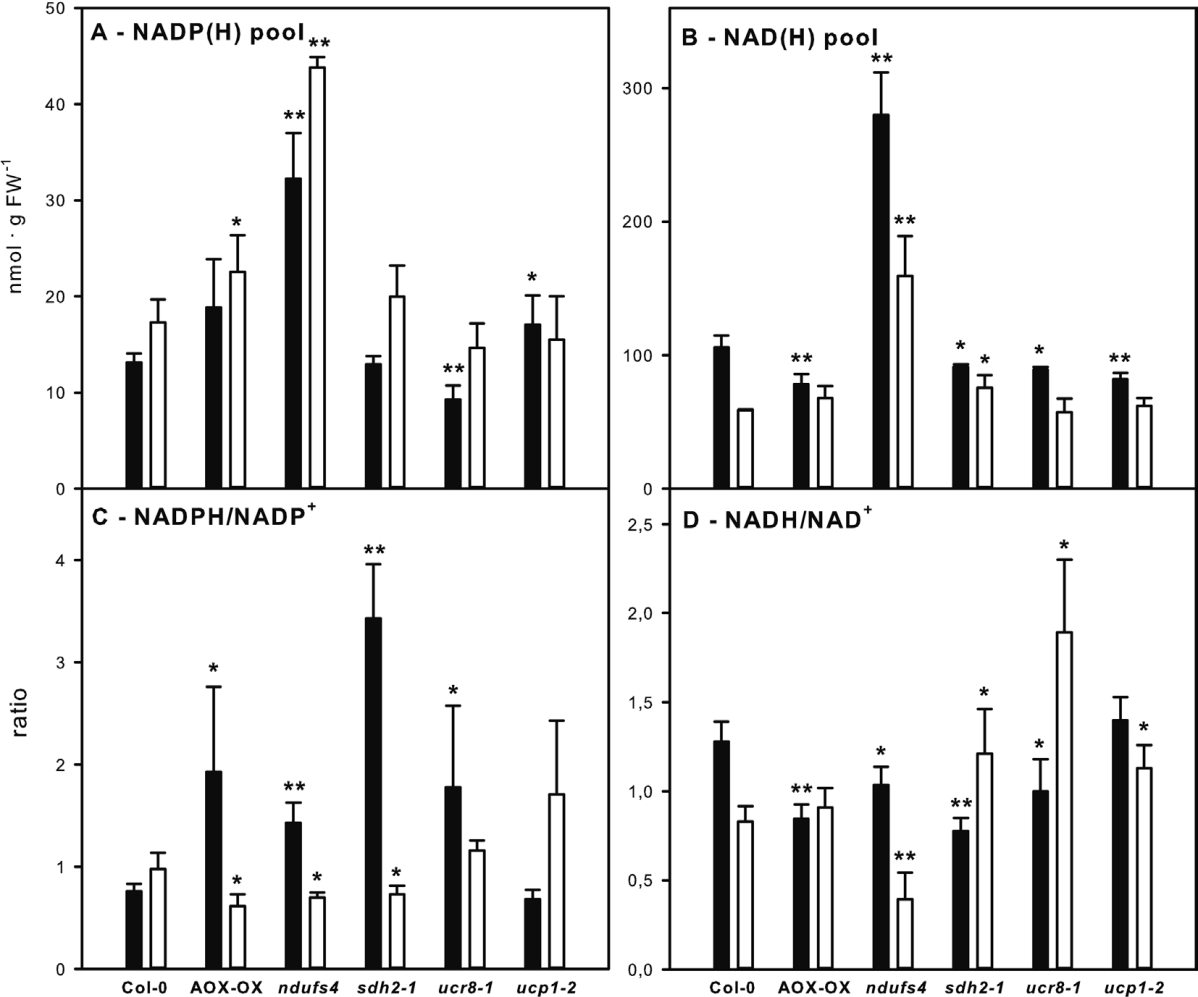
Analysis of reducing equivalent pools in mETC mutants. NADP(H) pool, i.e., NADPH + NADP^+^ **(A)**, NAD(H) pool, i.e., NADH + NAD^+^ **(B)**, NADPH/NADP^+^ ratio **(C)**, and NADH/NAD^+^ ratio **(D)** were determined in fully expanded leaves of 5-week-old plants at the end of the dark (black bars) and at the end of the light phase (white bars) in a 12-h light/12-h dark diurnal cycle. Genotypes are indicated below the bars. Data are means of four biological replicates per genotype and treatment ± SE. Significant differences to Col-0 in a pairwise t-test are indicated by asterisks with *: p < 0.05 and **: p < 0.005.

The observed increase in alternative respiration supports the idea that re-oxidation of NADPH and NADH, and hence, the redox state of the reducing equivalent pools, might be affected in the examined mutants. Therefore, we examined the NAD(H) and NADP(H) pools at the end of the light and at the end of the dark phase. In the *ndufs4* mutant, both NAD(H) and NADP(H) pools were increased more than two-fold relative to the wild type at both time points (**Figures 4A, B**). A comparable increase in NAD(H) pool size had already been observed for *ndufs4* by Kühn et al. (2015). The NAD(H) pool remained more oxidized in the *ndufs4* mutant than in the wild type, while the NADP(H) pool was more oxidized at the end of the light phase but remained more reduced at the end of the dark phase (**Figures 4C, D**). Similar to the *ndufs4* mutant, the reduction state of the NADP(H) pool was diminished in AOX-OX and *sdh2-1* mutants at the end of the light phase and was strongly elevated at the end of the dark phase in AOX-OX, *sdh2-1*, and *ucr8-1* (**Figure 4C**). In contrast to the other mutants, the reduction state of the NADP(H) pool in *ucp1-2* did not differ significantly from wild type. Consistently, all mutants except *ucp1-2* exhibited a diminished reduction state of the NAD(H) pool at the end of the dark phase (**Figure 4D**). Furthermore, the reduction state of the NAD(H) pool was increased two-fold in *ucr8-1* and by 30% in *sdh2-1* at the end of the light period (**Figure 4D**).

In summary, all genotypes that had exhibited an elevated rate of alternative respiration in the dark also showed an increase in the NADPH/NADP^+^ ratio and a decrease in the NADH/NAD^+^ ratio at the end of the dark phase (**Figures 4C, D**).

### The Analyzed mETC Mutants Show Clear Metabolic Phenotypes

Metabolite steady state levels of *sdhaf2* (Huang et al., 2013), *ndufs4* (Meyer et al., 2009), and *ucp1-2* (Sweetlove et al., 2006) were shown to differ distinctively from wild type in previous studies. We conducted metabolite profiling of 63 metabolites involved in major carbon and nitrogen metabolism at the end of the light period to obtain a comprehensive dataset that provides a blueprint of the metabolic status of the mutants in our growth conditions.

As described previously (Meyer et al., 2009), the *ndufs4* mutant showed an increase in the levels of all free amino acids between 3- and 10-fold compared to wild type (**Table 1**). Accumulation of glycine (36-fold) and serine (11-fold), which are produced during the photorespiratory C_2_ cycle, were most severe in *ndufs4*, followed by the glutamate-derived and stress-associated amino acids GABA (6.8-fold), proline (6.2-fold), and arginine (5.5-fold). In addition, the contents of free sugars and starch were also substantially elevated in *ndufs4* by 2-fold and 1.3-fold, respectively, as well as the TCA cycle intermediates fumarate and malate, which were increased by 1.6-fold relative to wild type (**Table 1**). Likewise, phosphorylated intermediates of the Calvin cycle and sucrose biosynthesis like RubP, Glc1P, Glc6P, and UDP-Glc were increased in *ndufs4* (**Table 1**), indicating an overflow of intermediates in the corresponding pathways. Tomato plants deficient in the TCA cycle enzyme aconitase exhibited a restricted flux through the TCA cycle that was associated with an increase in foliar adenylate, hexose phosphate, amino acid, and organic acid contents (Carrari et al., 2003). Furthermore, the *Aco-1* tomato mutants showed a redirection of carbon into non-structural carbohydrates and, concomitantly, increased photosynthetic productivity and yield (Carrari et al., 2003). While *ndufs4* also showed increased accumulation of sucrose and starch compared to wild type (**Table 1**), they are severely stunted (Meyer et al., 2009; Kühn et al., 2015). Metabolome analysis of *ndufs4* rather points to an extensive disturbance in all major metabolic pathways ranging from reducing equivalents, over amino acids to organic acids and sugars.

The metabolic phenotypes of the other analyzed mutants were less pronounced than that of *ndufs4*. In comparison to the wild type, the *ucp1-2* mutant exhibited a mild accumulation of almost all free amino acids as well as 20–30% higher starch and sucrose contents at the end of the light period, respectively (**Table 1**). No changes in reducing equivalent pools were observed in *ucp1-2* relative to wild type, which may reflect the comparatively mild metabolic phenotype of *ucp1-2* (**Figure 4**, **Table 1**). In support of this view, a previous metabolite profiling of *ucp1-2* grown in long-day conditions had not revealed any differences to the wild type (Sweetlove et al., 2006).

**Table 1:** Metabolite contents in Arabidopsis leaves at the end of the light phase.

Metabolite content (nmol · g FW^−1^)					
n = 4	Col-0	*ndufs4*	*sdh2-1*	*ucr8-1*	*ucp1-2*
ATP	101.6 ± 12.60	112.2 ± 2.39	96.42 ± 2.95	88.17 ± 1.88	103.9 ± 10.21
ADP	25.27 ± 1.27	**52.84** ± **1.75**	23.31± 1.13	25.06 ± 0.93	28.66 ± 1.62
AMP	8.69 ± 0.57	**17.53** ± **0.92**	8.63 ± 1.03	7.80 ± 0.05	8.54 ± 1.55
ADP-Glc	3.98 ± 0.98	2.22 ± 0.14	3.10 ± 0.06	5.23 ± 0.24	2.81 ± 0.18
UDP	6.46 ± 0.74	**17.66** ± **1.14**	6.09 ± 0.34	6.15 ± 0.50	6.24 ± 0.30
UDP-Glc	95.93 ± 6.19	**151.3** ± **9.83**	84.41 ± 11.14	115.7 ± 11.50	85.94 ± 1.08
RubP	3271.4 ± 82.98	**5520.2** ± **213.1**	**2835.8** ± **56.81**	3627.2 ± 204.2	2649.5 ± 311.9
3-PGA	504.6 ± 16.58	492.0 ± 32.88	361.8 ± 8.25	413.0 ± 5.47	464.6 ± 23.47
TrioseP	32.32 ± 8.45	31.05 ± 6.77	22.93 ± 2.06	21.84 ± 1.67	21.90 ± 3.77
Frc-1,6-bP	59.99 ± 1.09	63.10 ± 4.43	**50.37** ± **1.21**	**45.57** ± **3.27**	52.28 ± 6.19
Frc6P	183.4 ± 23.31	175.9 ± 24.20	118.7 ± 17.95	124.3 ± 9.65	130.3 ± 24.67
Glc6P	319.4 ± 2.39	**483.3** ± **61.69**	285.7 ± 33.66	**266.4** ± **12.43**	313.2 ± 9.53
Glc1P	49.77 ± 2.64	**65.06** ± **1.35**	47.11 ± 1.09	47.44 ± 1.60	46.61 ± 2.48
Glc-1,6-bP	10.54 ± 0.69	14.83 ± 1.27	13.08 ± 0.78	12.52 ± 1.49	16.17 ± 1.51
Man6P	49.63 ± 3.45	100.6 ± 6.20	49.10 ± 4.37	51.23 ± 3.71	45.76 ± 5.50
Suc6P	2.48 ± 0.28	1.65 ± 0.33	1.87 ± 0.20	2.37 ± 0.33	2.74 ± 0.40
Tre6P	0.33 ± 0.03	**0.57** ± **0.03**	**0.42** ± **0.01**	**0.52** ± **0.07**	**0.45** ± **0.02**
PEP	88.45 ± 3.64	107.4 ± 11.00	81.13 ± 2.03	84.31± 1.87	80.48 ± 6.76
Pyr	52.03 ± 5.62	**88.71** ± **12.12**	58.68 ± 5.00	41.27 ± 7.91	41.23 ± 6.45
Cit	11180.9 ± 527.9	11493.2 ± 213.4	9991 ± 621.7	10745.2 ± 1011.3	12506.0 ± 1580.1
Icit	1634.0 ± 212.4	1600.3 ± 122.1	1261.7 ± 55.66	1599.7 ± 116.7	1545.6 ± 238.4
OG	79.86 ± 5.82	73.83 ± 3.13	**55.43** ± **6.85**	**59.50** ± **2.63**	68.19 ± 10.83
Succ	232.4 ± 42.17	183.8 ± 2.76	**641.5** ± **71.62**	302.6 ± 6.57	280.1 ± 2.74
Fum	8020.8 ± 1286.4	**13112.3** ± **1186**	7295.2 ± 327.3	7818.9 ± 623.3	7736.6 ± 669.2
Mal	7157.9 ± 1162.7	**11164.5** ± **835.9**	6519.2 ± 118.7	7060.2 ± 569.7	7070.0 ± 799.4
Shi	43.39 ± 1.93	**55.38** ± **2.96**	**36.39** ± **0.76**	**31.84** ± **4.39**	32.62 ± 4.95
Metabolite content (nmol · g FW^−1^)					
n = 4	Col-0	*ndufs4*	*sdh2-1*	*ucr8-1*	*ucp1-2*
Asp	364.2 ± 57.85	**200.6** ± **23.97**	467.5 ± 37.91	**556.3** ± **13.91**	499.5 ± 65.33
Glu	730.4 ± 105.8	**2147.5** ± **129.7**	945.7 ± 64.44	**1150.9** ± **18.47**	985.1 ± 140.3
Asn	237.0 ± 41.54	**928.4** ± **58.74**	**341.7** ± **23.70**	**321.4** ± **14.45**	395.3 ± 12.87
Gln	1006.8 ± 203.17	**2491.6** ± **52.70**	**1517.4** ± **127.6**	1384.9 ± 99.57	**1793.6** ± **74.18**
Ser	430.3 ± 47.09	**4843.4** ± **864.5**	**774.1** ± **11.62**	515.2 ± 23.18	**617.8** ± **62.34**
Gly	107.4 ± 7.45	**3883.5** ± **364.1**	**209.9** ± **11.20**	**202.4** ± **27.82**	**230.6** ± **46.89**
Pro	65.15 ± 4.95	**401.7** ± **38.99**	86.67 ± 9.26	**99.82** ± **3.20**	**181.2** ± **35.90**
GABA	599 ± 142.1	**4062.4** ± **358.4**	750.9 ± 42.70	764.9 ± 25.56	**945.8** ± **78.77**
Arg	20.64 ± 1.33	**113.7** ± **7.47**	**37.24** ± **1.84**	**36.54** ± **2.41**	**38.56** ± **1.80**
His	14.82 ± 2.50	**52.18** ± **2.43**	19.16 ± 0.80	21.58 ± 1.59	**27.71** ± **1.40**
Lys	12.91 ± 1.90	**36.16** ± **4.57**	**18.26** ± **1.38**	**19.71** ± **1.61**	17.76± 0.85
Thr	125.2 ± 19.57	**348.8** ± **22.47**	137.6 ± 2.38	146.7 ± 11.03	169.8 ± 18.09
Leu	13.62 ± 1.60	**41.50** ± **1.73**	14.08 ± 0.70	16.53 ± 0.72	16.06 ± 1.43
Ile	15.09 ± 1.64	**47.81** ± **4.33**	16.95 ± 0.24	17.72 ± 0.77	**20.56** ± **1.19**
Val	48.51 ± 8.47	**147.0** ± **3.19**	65.03 ± 1.37	**68.70** ± **2.17**	**76.10** ± **3.34**
Ala	277 ± 41.04	**776.0** ± **119.5**	**445.6** ± **43.41**	**425.29** ± **11.57**	**527.1** ± **6.78**
Phe	31.75 ± 2.00	**83.88** ± **7.62**	**48.56** ± **2.43**	**53.53** ± **4.42**	**52.41** ± **6.64**
Tyr	6.82 ± 0.61	**23.15** ± **2.40**	9.35 ± 1.08	**9.37** ± **0.03**	7.65 ± 0.78
NADPH	9.60 ± 1.56	**14.58** ± **3.41**	8.30 ± 1.44	5.92 ± 2.10	6.49 ± 0.59
NADP	10.08 ± 0.27	**23.76** ± **2.28**	11.63 ± 2.12	**6.66** ± **0.95**	9.01 ± 4.19
NADH	28.16 ± 2.03	**53.29** ± **15.83**	**39.67** ± **3.40**	**41.38** ± **1.61**	32.44 ± 3.61
NAD	32.27 ± 1.48	**105.2** ± **19.06**	35.33 ± 6.71	18.56 ± 2.22	32.63 ± 1.96
NADPH + NADP^+^	17.28 ± 2.40	**43.82** ± **1.07**	19.93 ± 3.28	12.58 ± 2.72	15.50 ± 4.51
NADH + NAD^+^	58.74 ± 0.42	**159.2** ± **29.76**	**66.17** ± **11.49**	57.23 ± 10.23	61.91 ± 6.04
Metabolite content (nmol · g FW^−1^)					
n = 4	Col-0	*ndufs4*	*sdh2-1*	*ucr8-1*	*ucp1-2*
Total aa	4.11 ± 0.58	**20.66** ± **0.03**	**5.91** ± **0.19**	5.81 ± 0.57	**6.60** ± **0.20**
Glc	1.50 ± 0.20	**3.25** ± **0.48**	**2.10** ± **0.34**	1.27 ± 0.08	**0.86** ± **0.12**
Frc	0.19 ± 0.02	**1.38** ± **0.54**	**0.73** ± **0.07**	**0.51** ± **0.03**	0.36 ± 0.06
Suc	1.39 ± 0.14	**2.84** ± **0.57**	**1.61** ± **0.05**	**1.62** ± **0.07**	**1.93** ± **0.04**
Starch	59.36 ± 1.97	**82.74** ± **4.71**	63.22 ± 4.35	52.94 ± 2.19	**70.05** ± **2.71**

Fully expanded leaves from 5-week old plants were harvested 1 h before lights off in a 12-h light/12-h dark cycle. Data are means of four independent biological replicates ± standard error (SE). Significant differences to the wild type in a Student’s t-test with p < 0.05 are indicated in bold.

Amino acids and nucleotides are abbreviated according to standard three letter code, 3-PGA (3-phosphoglycerate), ADP-Glc (ADP-glucose), Cit (citrate), Frc-1,6-bP (fructose-1,6-bisphosphate), Frc6P (fructose-6-phosphate), Frc (fructose), Fum (fumarate), Glc-1,6-bP (glucose-1,6-bisphosphate), Glc1P (glucose-1-phosphate), Glc6P (glucose-6-phosphate), Glc (glucose), Icit (isocitrate), Mal (malate), Man6P (mannose-6-phosphate), OG (α-ketoglutarate), PEP (phosphoenolpyruvate), Pyr (pyruvate), RubP (ribulose-1,5-bisphosphate), Shi (shikimate), Suc6P (sucrose-6-phosphate), Suc (sucrose), Succ (succinate), total aa (total free amino acids), TrioseP (triose-phosphates), Tre6P (trehalose-6-phosphate), UDP-Glc (UDP-glucose).

In contrast, the *sdh2-1* mutant showed a distinct metabolic phenotype. We observed a three-fold accumulation of the SDH substrate succinate, while the levels of the upstream intermediate of the TCA cycle oxoglutarate were significantly reduced (**Table 1**). We also determined a 2-fold accumulation of glycine and a 1.8-fold accumulation of serine (**Table 1**), which are formed during recycling of carbon backbones from photorespiration. On top, N-rich amino acids like Asn, Gln, Arg, and Lys were about 50% elevated in *sdh2-1* compared to wild type (**Table 1**), indicating a specific shift in the C/N balance of the mutant.

The free amino acid pool was quite similarly affected in *ucr8-1*, although some of the differences were less pronounced than in *sdh2-1*, e.g., Gln and Ser (**Table 1**). Furthermore, NADH contents were elevated to a comparable extent in *sdh2-1* and *ucr8-1* (**Table 1**). While the specific accumulation of succinate was absent in *ucr8-1*, oxoglutarate contents were also similarly affected as in *sdh2-1* (**Table 1**).

### Altered Susceptibility of mETC Mutants Toward *C. higginsianum* Does Not Depend on SA

**Figure 5:**
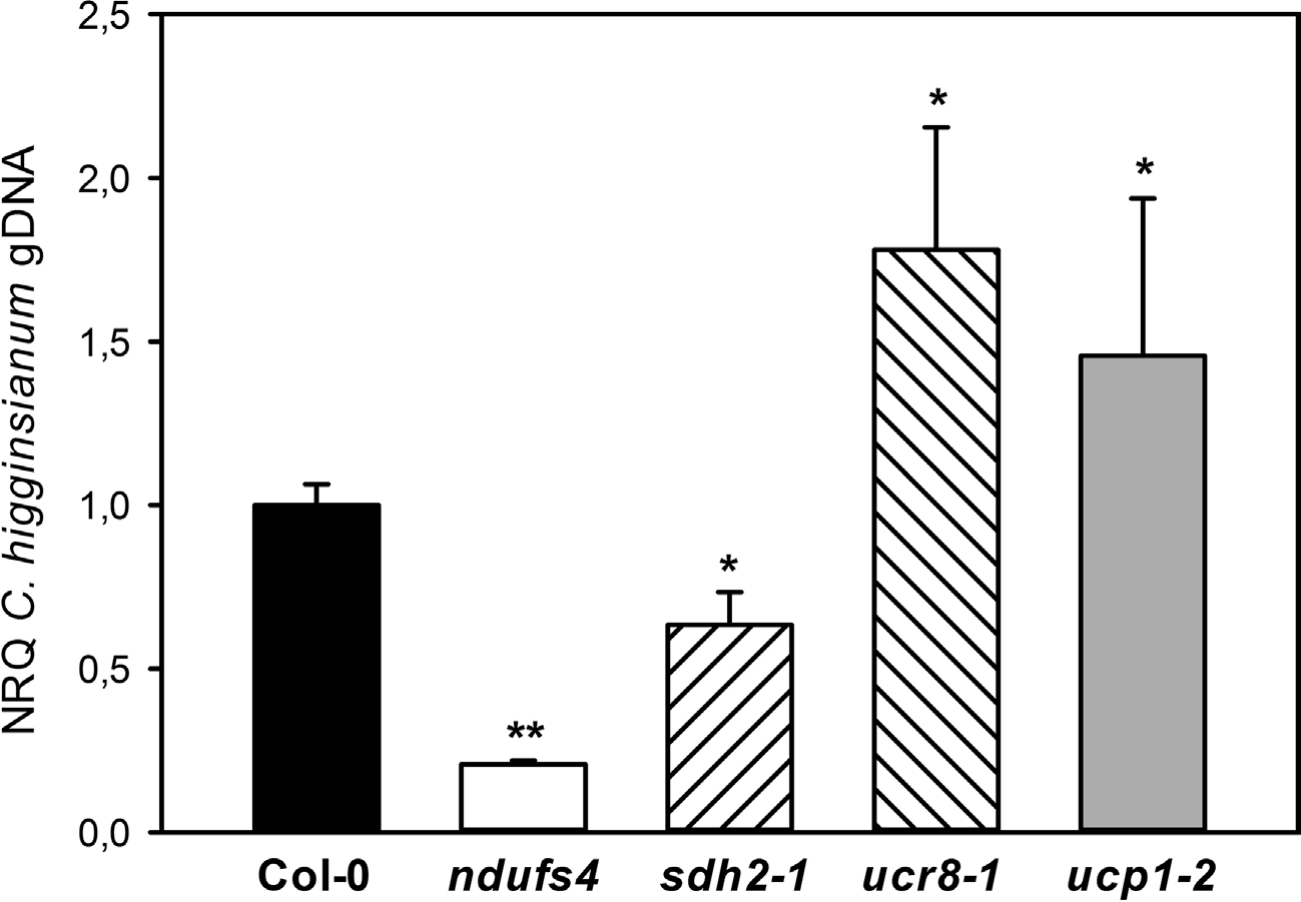
Susceptibility of mETC mutants toward *Colletotrichum higginsianum*. Five-week old plants grown in 12-h light/12-h dark cycles were spray infected with 2·10^6^ *Colletotrichum higginsianum* conidiospores/ml 1 h before the end of the light period. *C. higginsianum* genomic DNA was quantified in infected, fully expanded *Arabidopsis thaliana* leaves by qPCR at 3.5 days postinfection (dpi) and is given as normalized relative quantity (NRQ) relative to Col-0. Col-0 (black bar), *ndufs4* (white bar), *sdh2-1* (left hatched bar), *ucr8-1* (right hatched bar), *ucp1-2* (gray bar). The experiment was conducted at least five times, and data from one representative experiment with five biological and two technical replicates per genotype is shown ± SE. Significant differences to Col-0 in a pairwise t-test are indicated by asterisks with *: p < 0.05 and **: p < 0.005.

**Figure 6:**
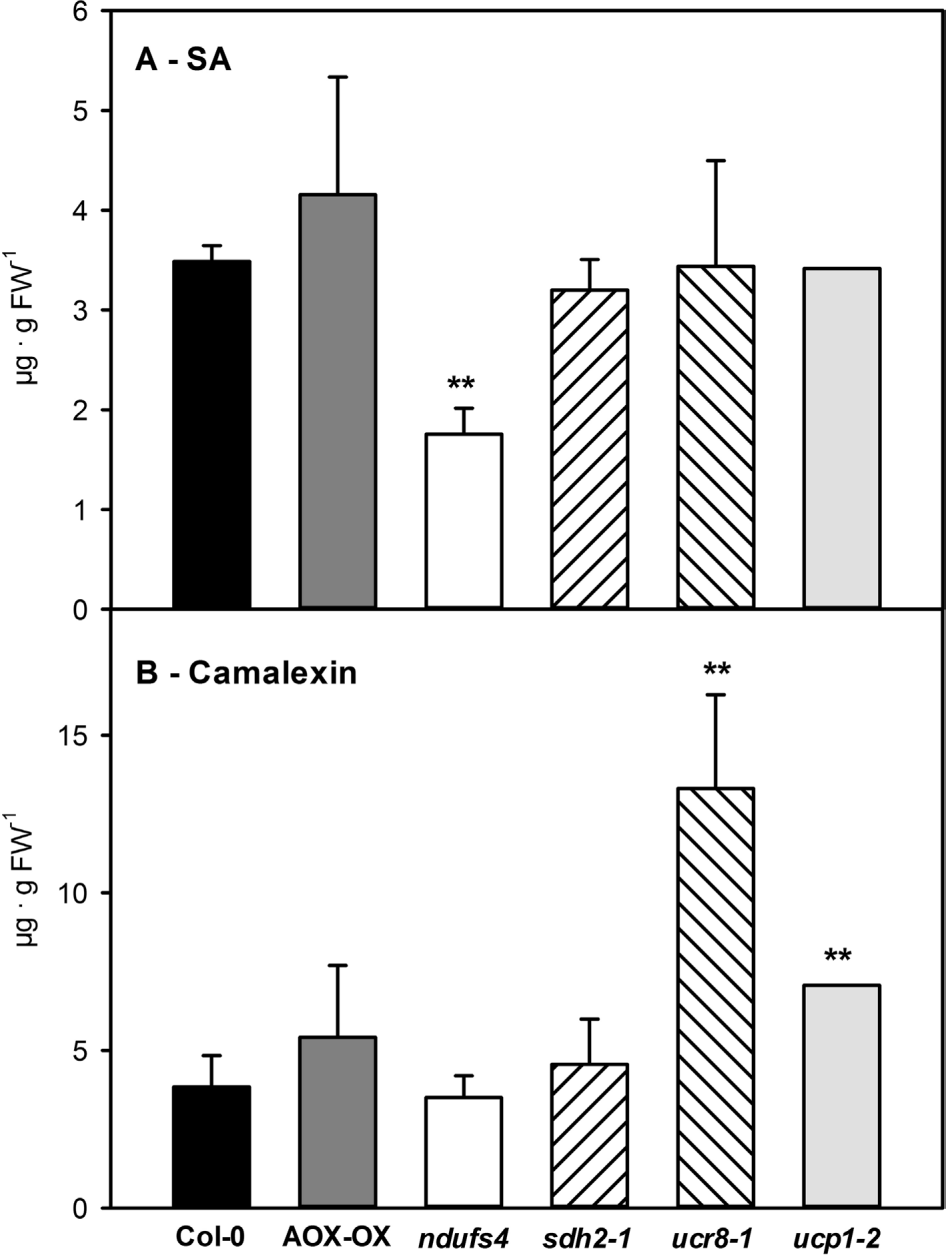
Accumulation of salicylic acid (SA) and camalexin in mETC mutants 2.5 days after inoculation with *Colletotrichum higginsianum* conidiospores. Five-week old plants grown in 12-h light/12-h dark cycles were spray infected with 2·10^6^ *C. higginsianum* conidiospores/ml 1 h before the end of the light period. Free SA **(A)** and camalexin **(B)** contents were determined in fully expanded leaves 2.5 dpi of Col-0 (black bars), AOX-OX (dark gray bars), *ndufs4* (white bars), *sdh2-1* (left hatched bars), *ucr8-1* (right hatched bars), *ucp1-2* (light gray bars). Data are means of four biological replicates per genotype and treatment ± SE. Significant differences to Col-0 in a pairwise t-test are indicated by asterisks with **: p < 0.005.

In summary, we have observed quite diverse changes in steady state metabolite levels, in the size and composition of the reducing equivalent pools as well as in the engagement of alternative respiration in the investigated mETC mutants. Therefore, we were curious to assess the interaction of the mutants with *C. higginsianum*. While differences in fungal colonization cannot be detected by qPCR during the biotrophic phase, determining fungal genomic DNA content in host leaves at 3.5 dpi was proven to represent differences in susceptibility during the necrotrophic colonization phase (Voll et al., 2012; Engelsdorf et al., 2013; Engelsdorf et al., 2017; Gebauer et al., 2017; Schmidt et al., in revision). In order to account for the reported growth phenotypes (Sweetlove et al., 2006; Meyer et al., 2009; **Figure S3**), fungal colonization was normalized to leaf area (as outlined in Engelsdorf et al., 2013). Interestingly, the *ndufs4* mutant proved to be highly resistant to *C. higginsianum*, while the *ucr8-1* and *ucp1-2* mutants were more susceptible toward the fungus (**Figure 5**). Unlike the intensively studied SDH1 mutant *dsr1* (Gleason et al., 2011; Belt et al., 2017), the *sdh2-1* mutant was significantly less susceptible toward pathogen infection than the wild type (**Figure 5**). Since SA accumulation and the response of the SA pathway were found to be dampened in *dsr1* mutants, our results prompted us to determine, if accumulation of free SA is affected in *sdh2- 1*. We quantified free SA contents in all investigated mutants at 2.5 dpi, which corresponds to the late biotrophic phase, when fungal spread is mostly limited to the infected epidermal cells in all genotypes and SA-dependent defense responses are thought to be effective. Alongside with SA, we quantified the accumulation of camalexin, the major phytoalexin in *Arabidopsis* (Zhao and Last, 1996). While camalexin was not detectable in mock controls, free SA contents did not show significant differences between genotypes and ranged between 0.11 ± 0.07 µg/g FW in *ndufs4* and 0.17 ± 0.03 µg/g FW in Col-0 wild type. Although SA and camalexin contents at 2.5 dpi were about 30-fold increased compared to mock controls, it should be noted that they were still approximately 10 times lower compared to heavily infected leaf tissue at 3.5 dpi (e.g., Engelsdorf et al., 2013). Most of the investigated genotypes showed comparable contents of SA and camalexin at 2.5 dpi, with three exceptions (**Figures 6A, B**). While *ucr8-1* and *ucp1-2* produced significantly more camalexin than the wild type (**Figure 6B**), the free SA content was significantly smaller in *ndufs4* compared to wild type (**Figure 6A**). Conversely, fungal colonization during the ensuing necrotrophic phase was elevated in *ucr8-1* and *ucp1-2*, despite increased camalexin production at 2.5 dpi, while fungal colonization was diminished in *ndufs4* despite reduced SA contents at 2.5 dpi. This discrepancy indicates that SA and camalexin contents at 2.5 dpi do not represent reliable predictors for compatibility at later stages of the infection. Most importantly, however, our results demonstrate that, unlike for *SDH1* mutants, SA accumulation is not hampered in *sdh2-1*.

### Only *ndufs4* Shows Elevated Basal Defense

**Figure 7:**
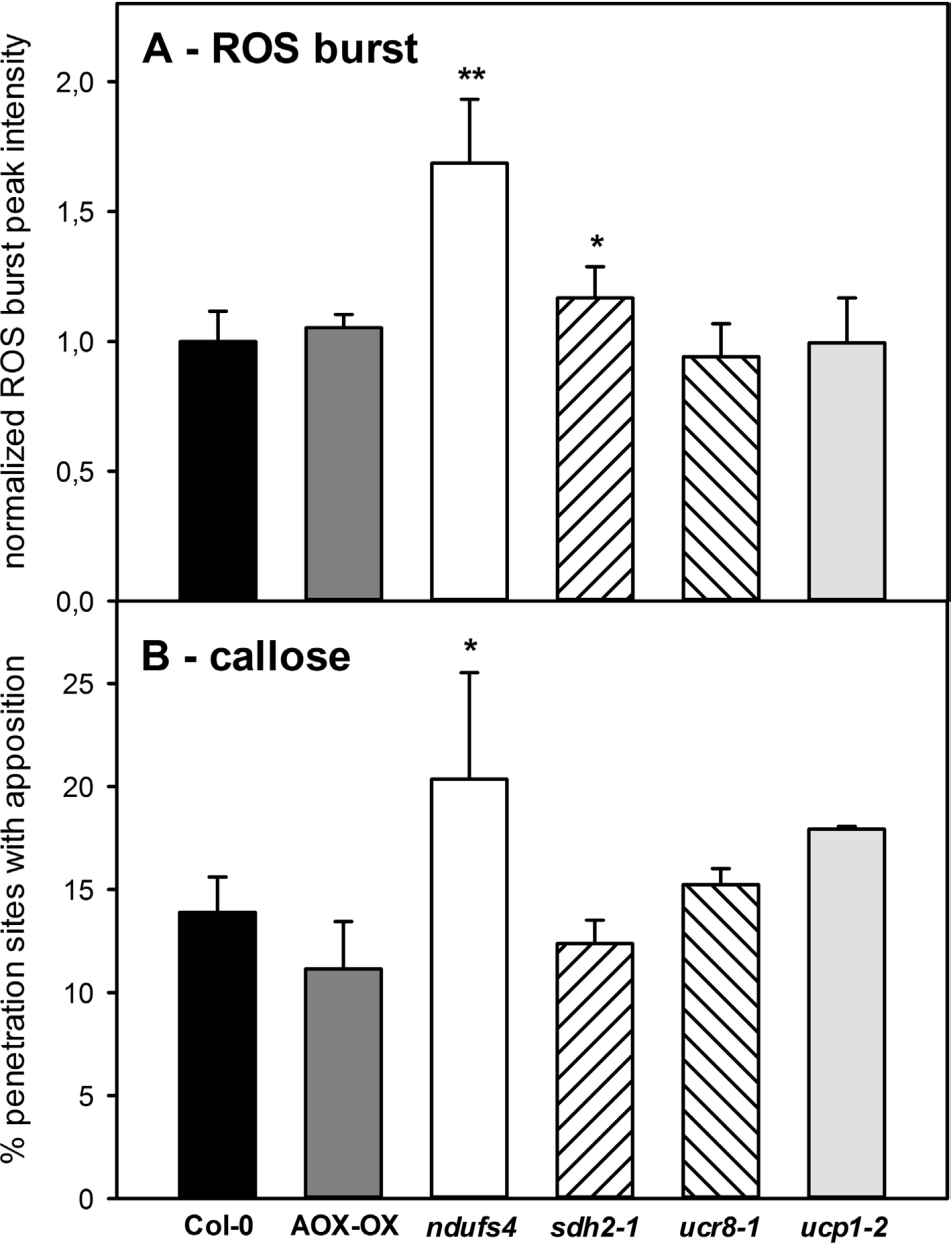
Basal defense responses in mETC mutants. To obtain a measure for the inducibility of basal defense responses in mETC mutants, the flg22-induced ROS burst (A) and the degree of callose apposition formation **(B)** were determined. Col-0 (black bars), AOX-OX (dark gray bars), *ndufs4 *(white bars), *sdh2-1* (left hatched bars), *ucr8-1* (right hatched bars), *ucp1-2* (light gray bars). Significant differences to Col-0 in a pairwise t-test are indicated by asterisks with *: p < 0.05 and **: p < 0.005. **(A)** Maximum ROS production in fully expanded leaves of 5-week-old plants was determined after challenge with 1µM flg22 by the luminol assay. Flg22-induced luminescence was recorded in three independent experiments with seven biological replicates each. Maximum luminescence was normalized to the average value of the respective wild-type controls in each experiment before means over all experimental replicates were calculated. Data are given ± SE. **(B)** Callose papillae formation in response to *Colletotrichum higginsianum* infection was quantified 2.5 dpi after *C. higginsianum* infection of *Arabidopsis thaliana* leaves with 2·10^6^ *C. higginsianum* conidiospores/ml. Four leaves of four independent plants per genotype were stained with aniline blue and for each leaf, at least 250 appressoria from different parts of the leaf blade were scored for the presence of callose papillae below fungal appressoria. In **(B)**, the fraction of appressoria exhibiting callose papillae is depicted. Data are means of four biological replicates ± SE.

Given these subtle differences in SA and camalexin accumulations in the mETC mutants, we tried to obtain other measures for the strength of the basal defense in the individual mutants by quantifying the ROS burst and the incidence of callose papillae at attempted penetration sites. The flg22-induced ROS burst was 1.6-fold increased in *ndufs4* and significantly elevated in *sdh2-1* but remained comparable for all other genotypes (**Figure 7A**). The *ndufs4* also showed an increased incidence of callose papillae underneath fungal appressoria; however, none of the other mETC mutants exhibited significantly increased callose formation (**Figure 7B**). As both the ROS burst and the formation of callose appositions were stimulated in *ndufs4*, this may indicate that fungal establishment is hampered at the pre-penetration stage in this mutant. If so, *C. higginsianum*–infected *ndufs4* leaves might exhibit less SA than the wild type because SA gets produced much stronger after successful penetration.

### Metabolic Signatures Correlate With Resistance to *C. higginsianum*

Except for *ndufs4*, we were unable to detect pronounced changes in the immediate response toward fungal challenge that would explain the observed interaction phenotypes. Along with *ndufs4*, three other mETC mutants had shown altered colonization with *C. higginsianum* compared to wild type during necrotrophic growth of the parasite. Since all four of these mutants were also affected in the energy balance of the mETC, we analyzed if the observed changes in steady state contents of the 63 analyzed primary metabolites can be linked to the susceptibility of the mutants. In order to exclude bias caused by differences in the progress of the interaction, we used data from non-infected leaves for the correlation analysis.

**Figure 8:**
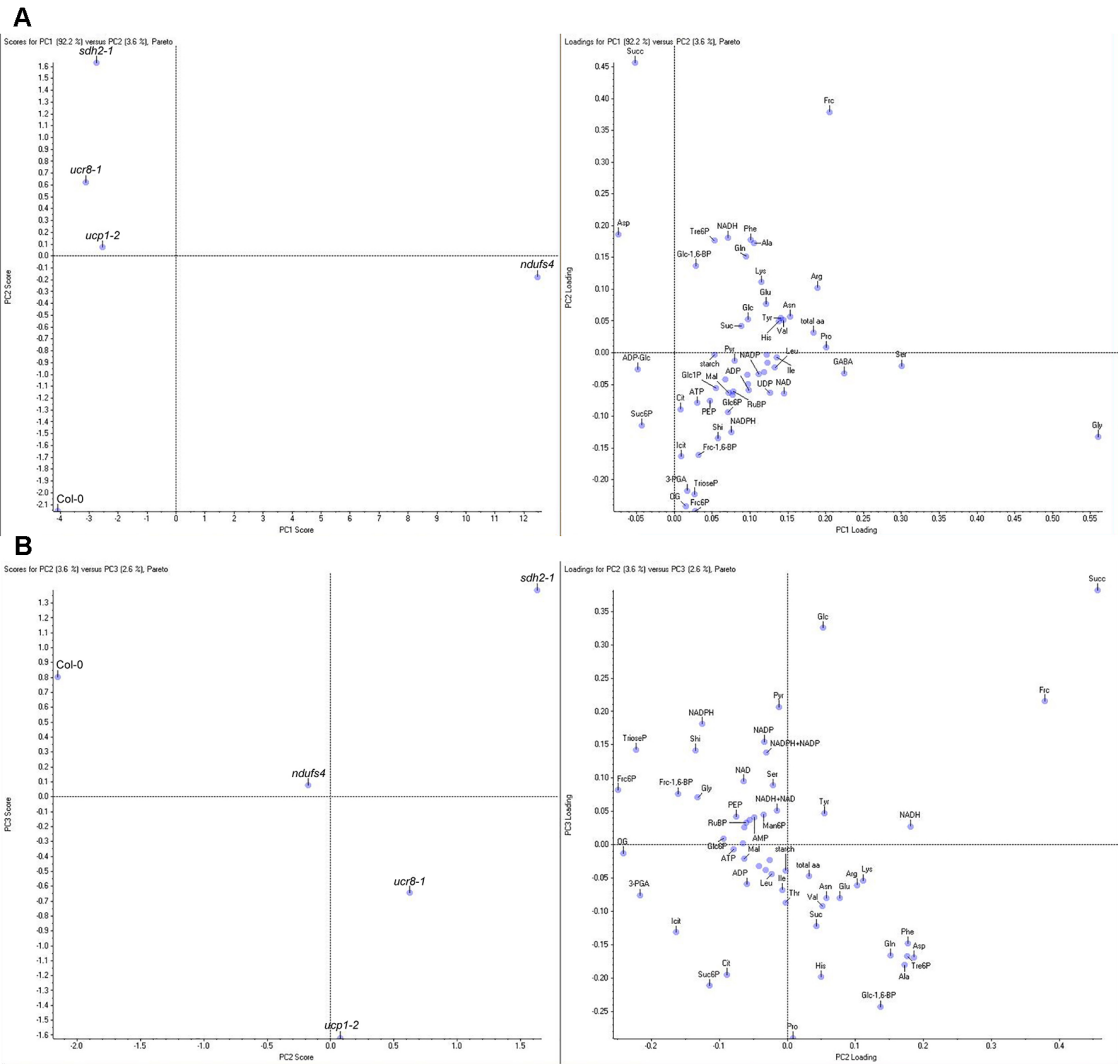
Principal component analysis (PCA) of metabolite data of mETC mutants. Metabolite contents were determined in fully expanded, uninfected leaves of 5-week-old plants 1 h before the end of the light period in a 12-h light/12-h dark cycle. Mean values of four biological replicates per genotype were subjected to PCA using the Pareto scaling algorithm of the Marker view software. The complete dataset with means ± SE is shown in **Table 1**. **(A)** Principal component 1 (PC1) plotted against PC2 (left panel) and corresponding loading plot (right panel). **(B)** PC2 plotted against PC3 (left panel) and corresponding loading plot (right panel). Amino acids and nucleotides are abbreviated according to standard three letter code, 3-PGA (3-phosphoglycerate), ADP-Glc (ADP-glucose), Cit (citrate), Frc-1,6-bP (fructose-1,6-bisphosphate), Frc6P (fructose-6-phosphate), Frc (fructose), Fum (fumarate), Glc-1,6-bP (glucose-1,6-bisphosphate), Glc1P (glucose-1-phosphate), Glc6P (glucose-6-phosphate), Glc (glucose), Icit (isocitrate), Mal (malate), Man6P (mannose-6-phosphate), OG (α-ketoglutarate), PEP (phospho*enol*pyruvate), Pyr (pyruvate), RubP (ribulose-1,5-bisphosphate), Shi (shikimate), Suc6P (sucrose-6-phosphate), Suc (sucrose), Succ (succinate), total aa (total free amino acids), TrioseP (Triose-phosphates), Tre6P (trehalose-6-phosphate), UDP-Glc (UDP-glucose).

**Figure 9:**
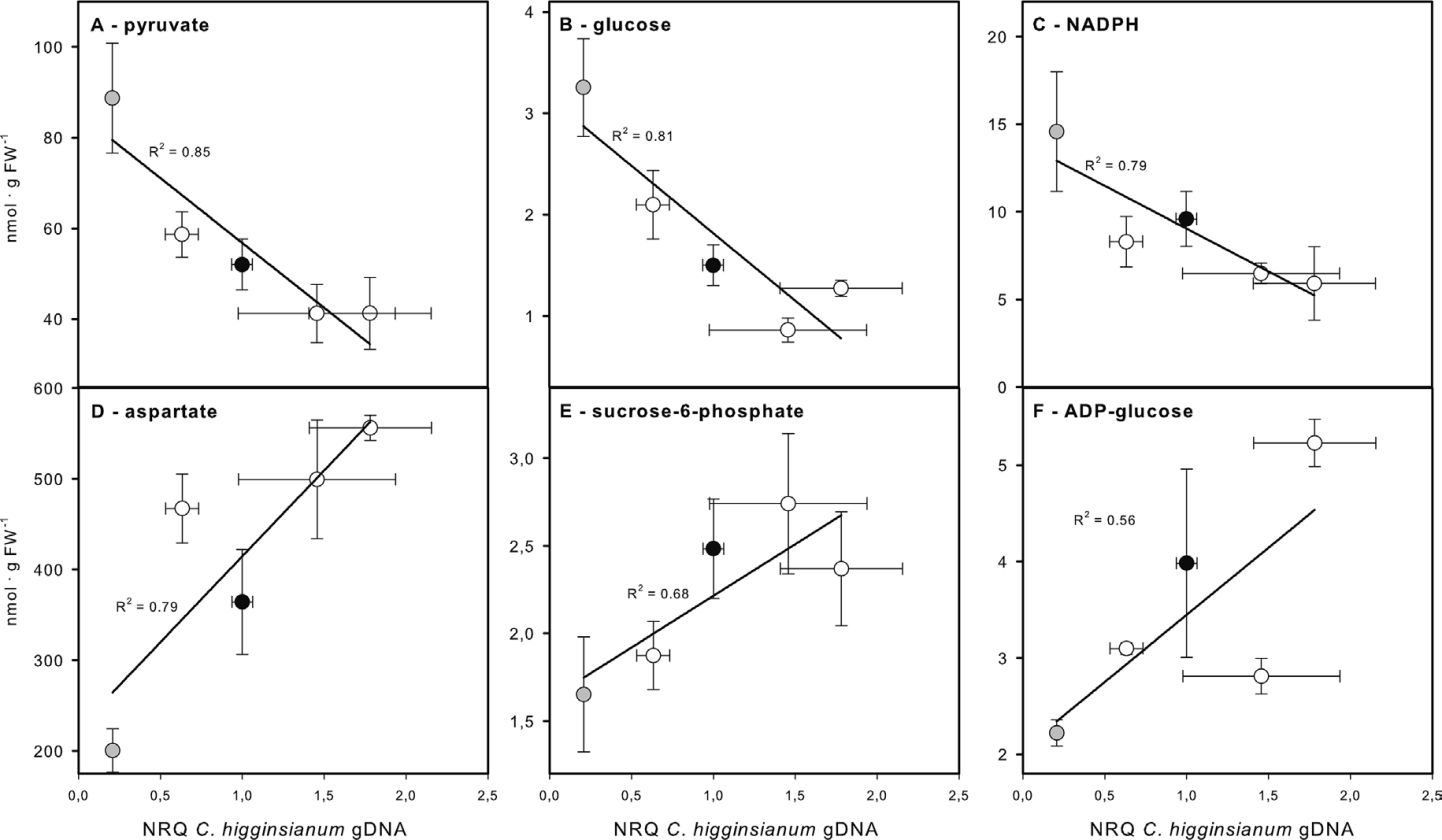
Correlation of metabolite contents with susceptibility toward *Colletotrichum higginsianum*. Correlation plots for the three metabolites showing the best negative **(A**–**C)** and positive **(D**–**F)** correlation with susceptibility (scored as normalized fungal genomic DNA content) are shown. **(A)** pyruvate, **(B)** glucose, **(C)** NADPH, **(D)** aspartate, **(E)** sucrose-6-phosphate, **(F)** ADP-glucose. Col-0 (black circles), *ndufs4* (gray circles), *sdh2-1*, *ucr8-1*, and *ucp1-2* (all white circles). The correlation coefficient R^2^ is given next to the individual regression lines. The complete correlation analysis for all metabolites is provided in **Table S1**. The results of the correlation analysis including data from AOX-OX can be found in **Table S2**. A correlation analysis without *ndufs4 *data points (gray circles) can also be found in **Table S2**.

A principal component analysis (PCA) of the entire profiling dataset revealed that more than 90% of metabolic variation between the genotypes can be explained by the metabolic phenotype of *ndufs4*, which loaded most strongly onto PC1 (**Figure 8A**). Both explaining around 3% of the metabolic variation, PC2 was capable of distinguishing *sdh2-1* from the other genotypes, while PC3 separates the two genotypes with increased susceptibility, *ucr8-1* and *ucp1-2*, from the rest (**Figure 8B**). The outcome of the PCA supports the idea that changes in the metabolite profile of the mETC mutants might correlate with compatibility, since the most resistant genotype *ndufs4*, as well as the two most susceptible genotypes, *ucr8-1* and *ucp1-2*, could be separated from the other genotypes by individual principal components. Therefore, we plotted metabolite content of non-infected leaves *versus* fungal colonization at 3.5 dpi and performed a correlation analysis with these data (**Table S1**). The contents of glucose, pyruvate, as well as the NADP(H) pool showed a strong negative correlation with susceptibility with R^2^ > 0.7 (**Figure 9**, **Table S1**). Typically, these metabolites indicate the availability of carbon building blocks and redox energy. Besides these strong candidates, only other intermediates that are indicators of carbon availability and photosynthetic performance (starch, Glc6P, Glc1P, Ser, Gly) as well as indicators of (redox) energy availability (ATP, AMP, NADH) exhibited a substantial negative correlation with susceptibility (R^2^ > 0.5; **Table S1**). On the other hand, only three metabolites showed a substantial positive correlation with susceptibility, namely, Asp, Suc6P, and ADP-Glc (**Figure 9**, **Table S1**). While Asp accumulates when nitrogen availability is high or under carbon limitation (e.g., Lam et al., 1994; Hanson et al., 2008), increased Suc6P and ADP-Glc levels may be associated with allosteric regulation of carbon allocation in favor of starch biosynthesis. The above listed metabolites still showed the strongest negative and positive correlations with susceptibility irrespective, if data from the *ndufs4* mutant were excluded (**Table S2**), demonstrating that the reported correlations do not depend on the inclusion of *ndufs4* in the analysis, which exhibited the most pronounced metabolic phenotype. When data from AOX-OX plants were included in the analysis, correlations of pyruvate contents and NADP(H) pool size with fungal colonization still prove to be robust (**Table S2**). However, adenine nucleotide pools, TCA cycle intermediates, and phosphorylated intermediates largely differed in AOX-OX from the other mutants analyzed (**Table S2**). Correlation coefficients calculated for these metabolite classes were substantially diminished upon inclusion of AOX-OX (**Table S2**), indicating that mutations affecting complexes I, II, or III have a different impact on the TCA cycle and energy metabolism than increased alternative respiration alone.

Taken together, our correlation analysis almost exclusively identified metabolites that are indicators for carbon allocation and the availability of reducing power and metabolic energy. While we had already identified diurnal foliar carbon turnover as a compatibility factor in the *Arabidopsis*–*C. higginsianum* interaction (Engelsdorf et al., 2013; Gebauer et al., 2017), the present analysis also identified pyruvate and the NADP(H) pool as determinants of compatibility, which can be taken as an indicator of mETC performance and integrity.

## Discussion

### Defects in *SDH1* and *SDH2* Lead to Similar Metabolic, But Contrasting Interaction Phenotypes

Mitochondrial succinate dehydrogenase, commonly designated as complex II, consists of four core and four accessory subunits in *Arabidopsis* (Huang and Millar, 2013). While the SDH1 subunit carries an FAD prosthetic group that is incorporated by SDH assembly factor 2 (SDHAF2; Huang et al., 2013), SDH2 contains 3 Fe-S clusters, and SDH3 and SDH4 bind the heme co-factor (Huang and Millar, 2013). To date, mutants affecting SDH1 and SDH2 functions have been studied (Elorza et al., 2004; León et al., 2007; Fuentes et al., 2011; Gleason et al., 2011; Huang et al., 2013; Belt et al., 2017). Functional studies are complicated by the fact that two *SDH1* and three *SDH2* genes exist in the *Arabidopsis* genome (Huang and Millar, 2013). While *SDH1-1* is the dominantly expressed SDH1 isoform in leaves (León et al., 2007), both *SDH2-1* and *SDH2-2* are expressed in leaves (Elorza et al., 2004). Interestingly, knockdown of SDH1 activity in *dsr1* point mutants, *sdh1-1* heterozygotes, and *sdhaf2* mutants resulted in different physiological and developmental phenotypes (León et al., 2007; Fuentes et al., 2011; Gleason et al., 2011; Huang et al., 2013; Belt et al., 2017). *sdh1-1* heterozygotes and one of the two characterized *sdhaf2* mutant alleles exhibited a comparable reduction of SDH activity by 30% and showed a similar defect in fertility (León et al., 2007; Huang et al., 2013). While *sdhaf2* mutants showed reduced root-tip respiration and elongation that are absent from *sdh1-1* heterozygotes (León et al., 2007; Huang et al., 2013), *sdh1-1* heterozygotes showed increased stomatal conductance and higher rates of photosynthesis (Fuentes et al., 2011), which was not observed for *sdhaf2* mutants (Huang et al., 2013). The *dsr1* mutant exhibits a reduction of SDH activity of 80% but is devoid of both phenotypes (Gleason et al., 2011; Belt et al., 2017), indicating that it is important how complex II function is disturbed. Instead, *dsr1* was shown to be more susceptible toward the necrotrophic fungi *A. brassicicola and R. solani*, as well as toward the hemibiotrophic bacterium *P. syringae *pv. *tomato*, which could be assigned to a reduced accumulation of SA (Gleason et al., 2011). SA accumulation was also hampered in *sdhaf2* mutants, albeit to a lesser extent (Belt et al., 2017). Consistently, SDH activity is less affected in *sdhaf2* mutants than in *dsr1* (Belt et al., 2017).

The *sdh2-1* mutant did not exhibit an obvious developmental phenotype, but given the fact that both *SDH2-1* and *SDH2-2* are expressed in leaves, it can be expected that SDH2 activity is substantially reduced in *sdh2-1* based on reporter gene assays (Elorza et al., 2004). In the frame of this study, we have observed a pronounced physiological phenotype of *sdh2-1* mutant leaves, ranging from increased alternative respiration (**Figure 3B**), a diminished reduction state of the NADP(H) pool in the light (**Figure 4**), (iii) a three-fold increased succinate accumulation (**Table 1**) as well as (iv) reduced susceptibility toward infection with the fungal hemibiotroph *C. higginsianum* (**Figure 5**). In addition, SA accumulation of *sdh2-1* during the biotrophic colonization phase was not altered compared to wild type (**Figure 6**), while the mutants *dsr1* and *sdhaf2* with a defect in SDH1 exhibited dampened SA accumulation and showed increased susceptibility toward fungal and bacterial pathogens (Gleason et al., 2011; Belt et al., 2017). These differences lead to the conclusion that processes at the SDH1, but not the SDH2 subunit, are required to trigger SA formation and resistance toward pathogens. Studies in animal mitochondria have demonstrated that ROS are generated at the SDH1 subunit of complex II (Yankovskaya et al., 2003; Quinlan et al., 2012). In the *sdh2-1* mutant, succinate oxidation to fumarate and concomitant FAD reduction can still take place at SDH1, while this is hampered in *dsr1* and *sdhaf2*. This suggests that the induction of ROS generation by SDH1 represents the trigger for enhanced SA synthesis.

### Metabolic Phenotypes of mETC Mutants Connect the Oxidation of Photorespiratory Glycine and Amino Acid Metabolism to Mitochondrial NADP(H) Status

The mutant *sdhaf2* accumulated approx. seven-fold more succinate than the wild type (Huang et al., 2013), while succinate accumulation was approximately half as severe in *sdh2-1* (**Table 1**). This further supports the idea that succinate oxidation by complex II is less affected in *sdh2-1*, which carries an intact SDH1 subunit, compared to *sdhaf2*, which has reduced SDH1 activity (Huang et al., 2013, Belt et al., 2017). Except for succinate, *sdh2-1* did not show an accumulation of organic acids as observed for *sdhaf2* (Huang et al., 2013), suggesting that the impact on the TCA cycle is weaker in *sdh2-1*. Nevertheless, the backup of serine and glycine, which are key indicators of the photorespiratory C_2_ cycle, was comparable in *sdh2-1* and *sdhaf2* relative to their respective wild types (**Table 1**, Huang et al., 2013), indicating that complete complex II activity is required to re-oxidize NADH generated during the oxidative decarboxylation of glycine in the mitochondrial matrix. In support of this assumption, barley mutants deficient in the mitochondrial glycine decarboxylase complex (GDC) showed increased cytochrome-dependent respiration compared to wild type and several physiological indications for a backup of reducing power in cytosol and chloroplasts (Igamberdiev et al., 2001). Besides the already discussed changes in glycine and serine, *sdh2-1* mutants were characterized by a comparatively mild accumulation of some free amino acids relative to the wild type (**Table 1**). An accumulation of free amino acids was largely absent from tomato plants with antisense repression of *SDH2-2* (Araujo et al., 2011), indicating that the impact of *SDH2* knockdown may depend on the level of residual activity and the species investigated.

Compared to *sdh2-1*, the accumulation of free amino acids was much more pronounced in *ndufs4* (**Table 1**). Free amino acid levels were increased in *ndufs4* between 3- and 10-fold compared to wild type, which is in agreement with previous results by Meyer et al. (2009). Accumulation of glycine (36-fold) and serine (11-fold) were most severe in *ndufs4*, followed by the glutamate-derived and stress-associated amino acids GABA (6.8-fold), proline (6.2-fold), and arginine (5.5-fold). Tobacco plants lacking a functional complex I exhibited a stimulation of nitrate assimilation and an increase in free amino acid content of more than two-fold (Dutilleul et al., 2005), which is consistent with the situation in *ndufs4* (**Table 1**). Since the assimilation of nitrate is an energy intensive process, in which 10 electrons are required to produce one molecule of glutamate, its stimulation might help to regenerate NAD^+^ in the cytosol when complex I is defective. Interestingly, the reducing equivalent pools are more reduced in *ndufs4* compared to wild type at the end of the dark phase, while nitrate reduction occurs during the light phase (for a comprehensive review see Stitt et al., 2002), which indicates that nitrate assimilation might indeed serve as a sink for reducing power in *ndufs4* during the light phase.

Furthermore, the massive backup of glycine and serine indicates that photorespiratory flux is hampered much more by limited NADH-regeneration in the mitochondrial matrix of *ndufs4* than in *sdh2-1* (see paragraph above). This is not surprising, since *ndufs4* lacks complex I activity, which directly oxidizes NADH (Meyer et al., 2009; Kühn et al., 2015), while NADH oxidation is not affected in complex II mutants (Huang et al., 2013). Vice versa, the reduction state of the cytosolic NAD(H) pool was increased in barley GDC mutants with hampered NADH re-oxidation in mitochondria (Igamberdiev et al., 2001).

However, nearly all quantified metabolites show a substantial accumulation in *ndufs4* compared to wild type (**Table 1**), which makes it difficult to disentangle immediate from indirect effects. In the light of the reduced leaf expansion of *ndufs4*, it can also not be ruled out that part of the observed differences to wild type is due to reduced vacuolization of mutant leaf cells.

The physiological phenotype of the *ucp1-2* mutant provides additional clues to the picture obtained from *ndufs4*. It has previously been shown that photorespiratory glycine oxidation is reduced three-fold in *ucp12*, but no substantial differences to the wild type were observed in a metabolite profiling approach comprising intermediates of major carbon and nitrogen metabolism (Sweetlove et al., 2006). In contrast to the previous study, we have observed a mild accumulation of glycine and serine and most free amino acids in *ucp1-2* compared to the wild type (**Table 1**). This may be due to the fact that we grew the plants in a 12-h light/12-h dark cycle, while Sweetlove et al. (2006) used long day–grown plants for their experiments. Nevertheless, our data on the diurnal accumulation of soluble sugars is fully consistent with the previous study, showing an increase in the sucrose/glucose ratio in the mutant (**Table 1**). Interestingly, the reducing equivalent pools of *ucp1-2* did not show any significant difference to wild type (**Table 1**).

Taken together, *ucp1-2* shows elevated contents of free amino acids, while reducing equivalents and major carbohydrates remain almost unaffected. In contrast, a substantial increase in free amino acids, sugars, and reducing equivalent pools were evident in *ndufs4* (see above). This difference may indicate that decreased photorespiratory flux and increased nitrate assimilation are primary responses toward a limitation in mitochondrial NADH oxidation, while the effects on reducing equivalent pools and major carbohydrate metabolism are of secondary nature. In addition, one has to bear in mind that loss of *UCP1* will exert a much milder restriction on mitochondrial NADH oxidation compared to a loss of complex I in *ndufs4*, since UCP1 will only be engaged in physiological circumstances when flux through the mETC is hampered.

Similarly, the *ucr8-1* mutant showed a comparatively mild, but different physiological phenotype compared to *ucp1-2*. Besides showing a substantial reduction in growth and gross dark respiration, the degree of alternative respiration as well as glycine contents and the levels of several other amino acids were markedly increased in *ucr8-1* (**Table 1**). In addition, the reduction states of the NADP(H) and the NAD(H) pool were quite severely affected compared to other mETC mutants (**Figure 4**). As further supported by PCA analysis, the overall metabolic phenotype of *ucr8-1* is very similar to *sdh2-1* and *ucp1-2* in several respects, which corroborates that the defect in *ucr8-1* is associated with the mETC.

### Pyruvate and NADP(H) Pool Size Are Metabolic Determinants of Compatibility

Our study was set out to investigate if metabolic processes associated with the integrity of the mETC can affect compatibility. To this end, we have selected a diverse set of mutants that share i) reduced total dark respiration and ii) elevated alternative respiration but iii) are affected in different functions of the mETC. Besides previously characterized mutants affecting complex I, complex II, and the uncoupling protein UCP, we included the newly identified complex III knock-down mutant *ucr8-1*. Similar to what is published for the only *Arabidopsis* mutant affected in complex III activity known to date, *ppr40-1* (Zsigmond et al., 2008), *ucr8-1* exhibited substantially diminished total respiration but elevated alternative respiration. Interestingly, *ucr8-1* as well as *ppr40-1* retain substantial complex III activity, indicating that complex III loss-of-function mutants might not be viable.

More than a dozen of the analyzed metabolites showed a negative correlation with fungal colonization, i.e., a negative correlation with susceptibility (**Table 1**, **Figure 9**). Interestingly, these metabolites almost exclusively comprised marker metabolites for carbon availability and photosynthetic performance, like glucose, starch, Glc6P, Glc1P, serine, and glycine (**Table 1**, **Figure 9**). Consistently, a previous study of our group had already indicated that overall carbon availability correlates with resistance toward *C. higginsianum* (Engelsdorf et al., 2013). In addition, the present study now identified that pyruvate contents and the NADP(H) pool size correlated strongly (R^2^ > 0.7), while NADH and adenine nucleotide pool sizes correlated substantially (R^2^ > 0.5) with resistance (**Figure 9**, **Table S1**). When data from AOX-OX plants were included in the analysis, correlations of pyruvate contents and NADP(H) pool size prove to be robust (**Table S2**). However, adenine nucleotide pools, TCA cycle intermediates, and phosphorylated intermediates largely differed in AOX-OX from the other mutants analyzed (**Table S2**), which made it necessary to exclude AOX-OX from the subsequent PCA analysis. A PCA with the metabolite dataset had shown that *ndufs4* exhibits the most pronounced metabolic phenotype of all investigated mutants (**Figure 8**), which is also characterized by a pronounced accumulation of all abovementioned marker metabolites (**Table 1**). In addition, pre-penetration defenses like the ROS burst and the formation of callose appositions were stimulated in *ndufs4*, which may indicate increased efficiency of the basal defense response in *ndufs4*. It is difficult to judge if a stimulation of basal defense somehow relates to the reported metabolite levels. Excluding the data from *ndufs4* from the correlation analysis leads to the same results (**Table S2**), indicating that the outcome of the analysis does not solely depend on the influence of one single genotype that exhibits improved pre-penetration defense.

The pools of reducing equivalents and adenine nucleotides might increase when a limitation in the regeneration of oxidized NAD(P)^+^ or ADP as acceptors for pETC and mETC is evident. In this light, resistance of the mETC mutants correlated with the limitation in NAD(P)^+^ or ADP regeneration. However, these metabolic limitations are associated with a surplus in reducing power in mitochondria. Although we have not examined subcellular pools of redox equivalents in this study, it is known that an over-reduction of mitochondrial NADP(H) and NAD(H) pools will be partially transferred to the cytosolic pools by the export of the organic acids malate and citrate, which are termed the malate and citrate valves (see Igamberdiev and Bykova, 2018 for a recent review). In the cytosol, malate and citrate can be utilized to produce reducing equivalents by their oxidation to oxaloacetate and oxoglutarate, respectively. Therefore, it can be expected, that besides the mitochondrial pools, also the cytosolic NADP(H) and NAD(H) pools remain in a more reduced state, especially in *ndufs4* mutants. *Arabidopsis* mutants lacking cytosolic NADP-malic enzyme (ME) were more susceptible against *C. higginsianum*, demonstrating that extra reducing power in the cytosol is required to support an effective defense reaction *versus C. higginsianum* (Voll et al., 2012). In parallel, elevated NADPH levels in the mitochondrial matrix will increase the reduction state of specific thioredoxins, which will lead to redox activation of AOX (Gelhaye et al., 2004). Reduced AOX can then be further activated covalently by pyruvate and other β-keto acids (e.g., Selinski et al., 2017 and literature cited therein). This activation of alternative respiration helps to ameliorate the congestion of electron flow in the mETC and thereby prevents oxidative stress that might counteract an effective host defense response.

Based on our data, we cannot identify which metabolic signals lead to a stimulation of the defense response of the plant and what kind of molecular sensors are involved. This will be an interesting subject for future studies.

## Data Availability Statement

All datasets generated for this study are included in the manuscript/**Supplementary Files**.

## Author Contributions

LV designed the research with input from AV. CM, SG, TE, and AV conducted the experiments. CM, SG, AV, TE, and LV discussed the results. LV interpreted the results and wrote the manuscript.

## Conflict of Interest

The authors declare that the research was conducted in the absence of any commercial or financial relationships that could be construed as a potential conflict of interest.

## Supplementary Material

Table S1 Correlation of fungal proliferation with individual metabolite contents in the investigated genotypes.

Table S2 Sensitivity analysis of the correlations between fungal proliferation and metabolite contents.
