# Supplementary material for: Deficiencies in the Mitochondrial Electron Transport Chain Affect Redox Poise and Resistance Toward Colletotrichum higginsianum
Source: Front Plant Sci. 2019 Oct 17;10:1262. doi: 10.3389/fpls.2019.01262 (PMC6812661; doi:10.3389/fpls.2019.01262)
Supplement: Supplementary file 2 [file DataSheet_2.pdf]

Supplemental Material - McCollum et al.

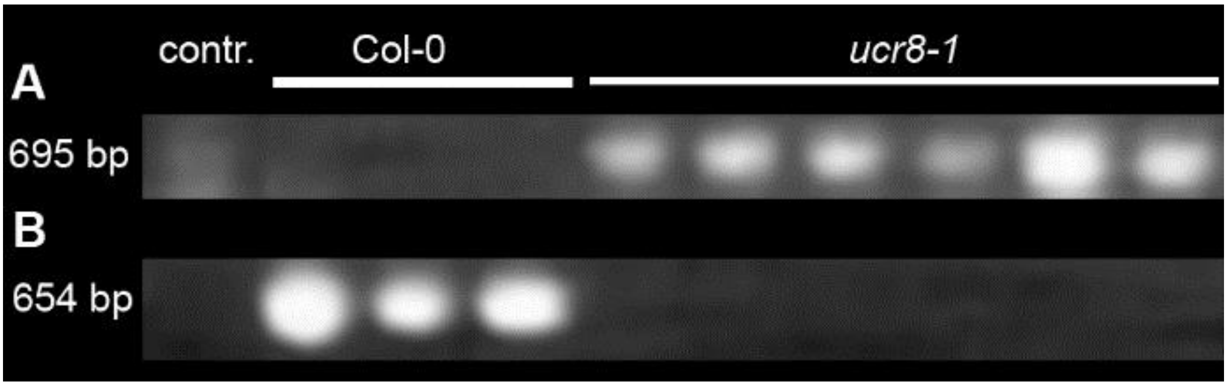

Figure S1. Genotyping of the *ucr8-1* mutant by PCR.

A 695 bp fragment of the T-DNA insertion allele was amplified using the oligonucleotide primers SALK_022835-RP and LBb1 (A), while a 654 bp fragment of the wild type allele was amplified employing the oligonucleotide primers SALK_022835-RP and SALK_022835-LP (B). Per lane, three wild type control (Col-0) and six *ucr8-1* samples were loaded next to a no template control (contr.). Please see the materials and methods part for oligonucleotide sequences.

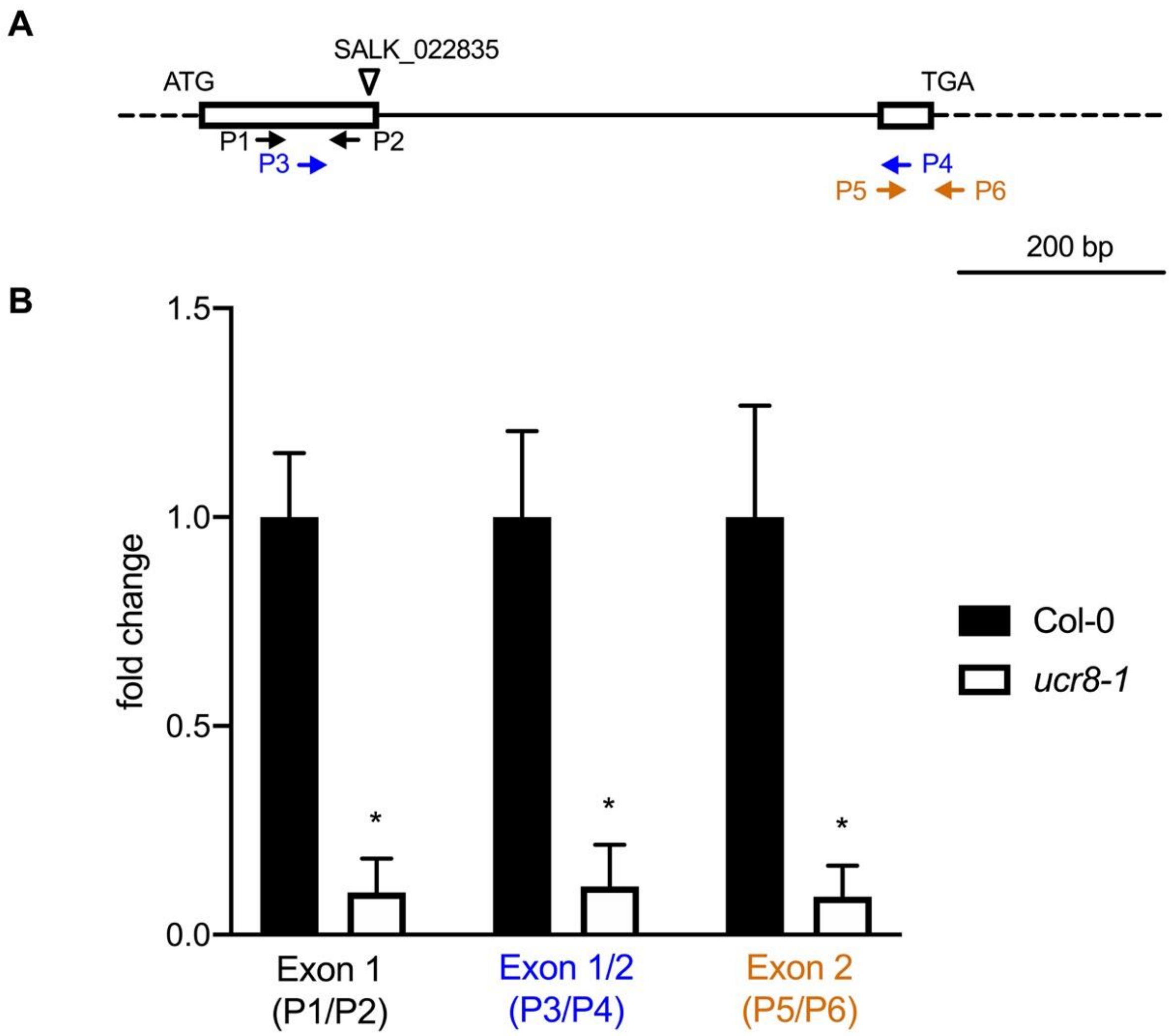

Figure S2. Characterization of *ucr8-1* by qRT-PCR.

(A) *UCR8* gene model indicating the T-DNA insertion site in *ucr8-1* (SALK_022835; triangle) and PCR primers used (arrows). Specific primer combinations were used to amplify sequences in exon 1 (P1/P2), exon 1/2 (P3/P4) and exon 2 (P5/P6), respectively. Exons are represented by boxes, introns by lines and untranslated regions by dashed lines. The size bar indicates 200 bp. (B) The normalized relative quantity of *UCR8* sequences was determined using the primer combinations indicated above and *ACTIN2* as reference gene. Values are shown as fold change to Col-0 and error bars indicate SD (n=4). Asterisks indicate statistically significant differences to Col-0 according to Student's t- test ($p < 0.05$).

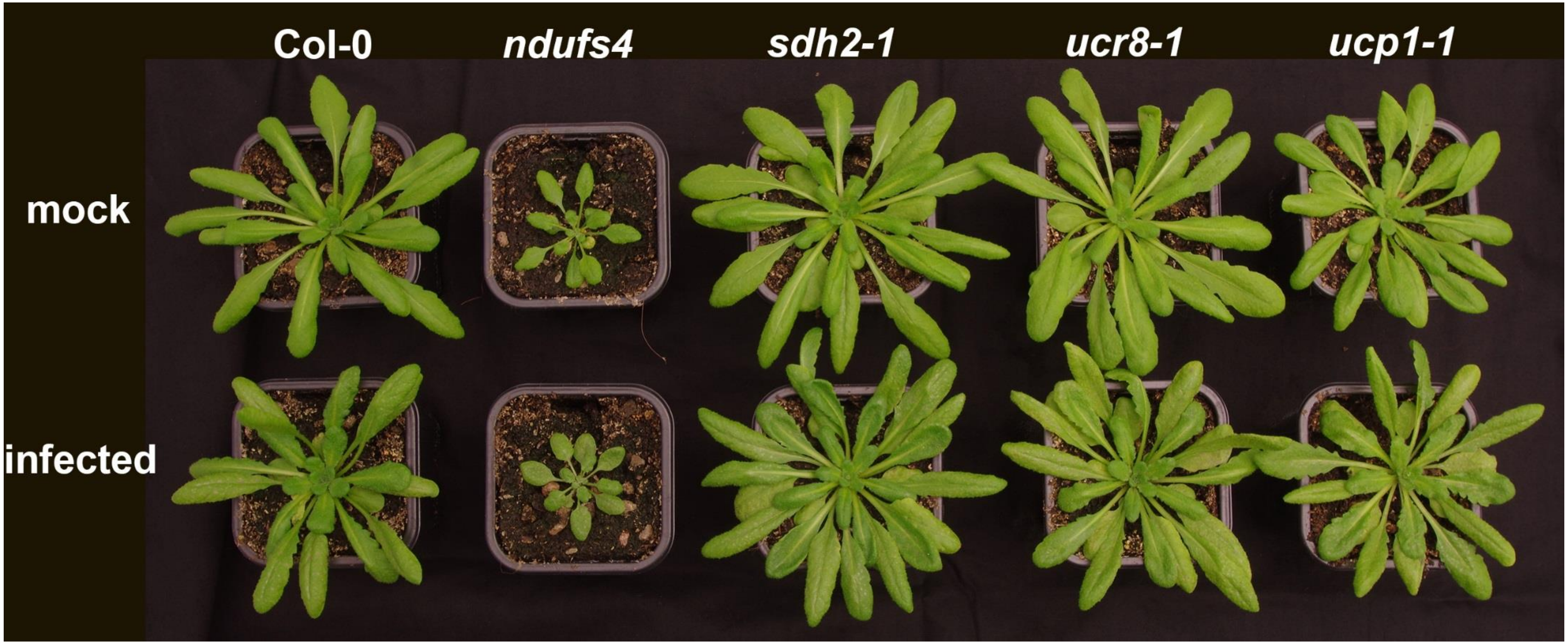

Figure S3. Growth and interaction phenotypes of the investigated Arabidopsis genotypes at 3.5 days after infection with *C. higginsianum.* Plants of the indicated genotypes were spray infected with $2 \cdot 10^6$ *C. higginisanum* conidiospores/ ml 4 h before the end of the light period (bottom row). For comparison mock control plants are depicted in the top row.
